# Revealing the Full Potential of Glycolated Mixed Ionic-Electronic Semiconductors – Symmetric Monomer Polymerization to Boost Electrochemical Transistor Performance

**DOI:** 10.1021/jacs.5c19024

**Published:** 2026-02-23

**Authors:** Lize Bynens, Paola Mantegazza, Adam Marks, Yeongmin Park, Arwin Goossens, Stefania Moro, Tyler J. Quill, Garrett Lecroy, Christina Cheng, Arianna Magni, Laurence Lutsen, Jochen Vanderspikken, Simon E. F. Spencer, Koen Vandewal, Alberto Salleo, Giovanni Costantini, Wouter Maes

**Affiliations:** † Hasselt University, 54496 Institute for Materials Research (imo-imomec), Design & Synthesis of Organic Semiconductors (DSOS), Martelarenlaan 42, B-3500 Hasselt, Belgium; ‡ 168164 imec, imo-imomec, Wetenschapspark 1, B-3590 Diepenbeek, Belgium; § School of Chemistry, 1724 University of Birmingham, Edgbaston, Birmingham B15 2TT, United Kingdom; ¶ Department of Materials Science and Engineering, 6429 Stanford University, Stanford, California 94305, United States; # Hasselt University, Institute for Materials Research (imo-imomec), Organic Optoelectronics (OOE), Martelarenlaan 42, B-3500 Hasselt, Belgium; ° Department of Statistics, 2707 University of Warwick, Coventry CV4 7AL, United Kingdom; ± School of Physics and Astronomy, University of Birmingham, Edgbaston, Birmingham B15 2TT, United Kingdom

## Abstract

Organic electrochemical transistors (OECTs) enable the transduction of ionic signals into electronic outputs, positioning them as ideal candidates for next-generation sensing and (bio)­signal processing applications. Recent years have witnessed the development of various OECT channel materials, affording insights into structural fine-tuning to achieve optimal performance and/or stability. However, homocouplings, commonly present in alternating conjugated polymers, have largely been overlooked. This study investigates the effect of homocoupling on OECT materials by employing two synthesis methods – standard Stille polymerization and an alternative symmetric approach – to create the p-type enhancement-mode benchmark polymer pgBTTT. The impact of homocoupling, and its absence, is studied by comparing the bulk properties of the two polymers and evaluating their respective OECT metrics. The new, homocoupling-free polymer exhibits a notably improved OECT performance (μC*), mainly due to an average 3-fold increase in electronic mobility (μ).

## Graphical abstract

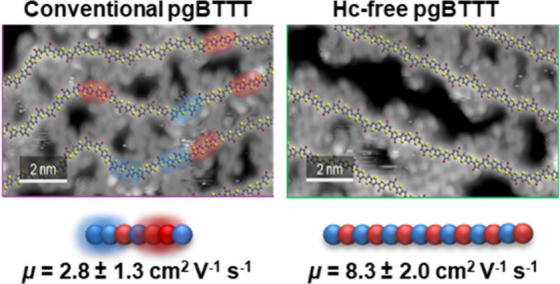

## Introduction

State-of-the-art alternating semiconducting copolymers are traditionally synthesized via established cross-coupling protocols such as Stille and Suzuki polymerization.^1–3^ The resulting alternating structure is most often simply assumed based on the flawless selectivity of the two complementary functionalized comonomers. In practice, however, two identical monomers can react instead, producing homocoupling defects. This causes the obtained structure to deviate from the perfectly alternating one.^4–8^ Since the occurrence of homocoupling depends on many factors and does not appear consistently, these structural defects hamper the reproducibility across different polymer batches, posing a challenge to the commercial viability of polymeric semiconductor applications. Furthermore, homocoupling defects may induce performance limitations,^1^ as shown for organic photovoltaics and photodetectors.^5,9–14^

The occurrence and impact of structural defects in organic electrochemical transistor (OECT) materials remain largely unexplored. OECTs have garnered significant attention due to their exceptional sensitivity and selectivity, coupled with their compatibility with soft and flexible substrates.^15^ These devices offer a unique interface between ionic and electronic conductors, enabling the transduction of ionic (bio)­signals into electronic outputs.^16^ Significant efforts have been directed at increasing the performance of p-type OECTs in enhancement-mode, where the application of a gate potential is required to dope the channel material with positive electronic charge carriers (holes).^17,18^ The enhancement-mode operation allows for a high transistor gain, low-power operation, and high sensitivity, making it a sought-after configuration for sensing applications.^19,20^ In this context, the choice of an appropriate channel material plays a pivotal role in determining the overall device performance and operational characteristics.^21^ Recent years have witnessed the development of many new, often intricate OECT channel materials.^21–28^ Among these, glycolated organic mixed ionic-electronic conductors (OMIECs) have shown significant promise in achieving high performance in p-type OECTs because of their volumetric electrochemical charging capabilities.^17,22,24,25,29^ However, studying performance-limiting morphological and structural factors in these materials presents challenges due to the complex interplay between ionic and electronic charges within an OECT.^30,31^ Computational modeling studies can contribute to our understanding but typically assume perfectly alternating polymer backbones, which may not accurately reflect experimentally realized materials.^11,32^ As OECT performance continues to advance, attention should be directed toward the reproducibility and structural fidelity of these complex polymers. Structural defects can obscure structure–property relationships and hinder meaningful comparison between experimental and theoretical studies. It is therefore essential to ensure that the synthesized material precisely reflects its intended molecular structure in order to obtain reproducible device characteristics and accurate structure–property relationships.

To achieve a truly homocoupling-free polymer, alternative synthetic methods are required. Our study focuses on the homocoupling-free synthesis of the benchmark p-type enhancement-mode OECT material pgBTTT (poly­[4,4′-bis­(2-(2-(2-methoxyethoxy)­ethoxy)­ethoxy)-[2,2′-bithiophen]-5,5′-diyl]-*alt*-[thieno­[3,2-*b*]­thiophene-2,5-diyl]), first reported by Hallani et al.^33^ pgBTTT consists of glycolated bithiophene (gBT) and unsubstituted thieno­[3,2-*b*]­thiophene (TT) units, which in theory should alternate regularly. The standard Stille polymerization employed in the synthesis of pgBTTT and numerous other semiconducting polymers involves coupling a distannylated monomer to a dibrominated one using a palladium (Pd) catalyst (Scheme 1).^1^ This strategy is widely applied in academic settings because of its mild reaction conditions and the reasonably high molar masses it affords.^1^ However, this polymerization route introduces homocoupling defects within the polymer backbone, as recently illustrated for alkoxy-substituted pBTTT analogs (Scheme S3).^11,34^ While it is known that these side reactions occur and thus most OECT channel materials contain homocoupling defects, this aspect is generally disregarded when interpreting OECT results. On the other hand, molar mass has been established as a critical parameter with substantial influence on the performance of both p- and n-type OECTs.^35–39^ For p-type OECTs, it has been reported that increasing the molar mass of the channel material enhances mobility and hence overall performance, up to a system-specific threshold, after which it decreases again.^38,39^ However, different molar mass fractions may contain a different number of defects, adding to the uncertainty of which parameter truly determines performance.

**Scheme 1:**
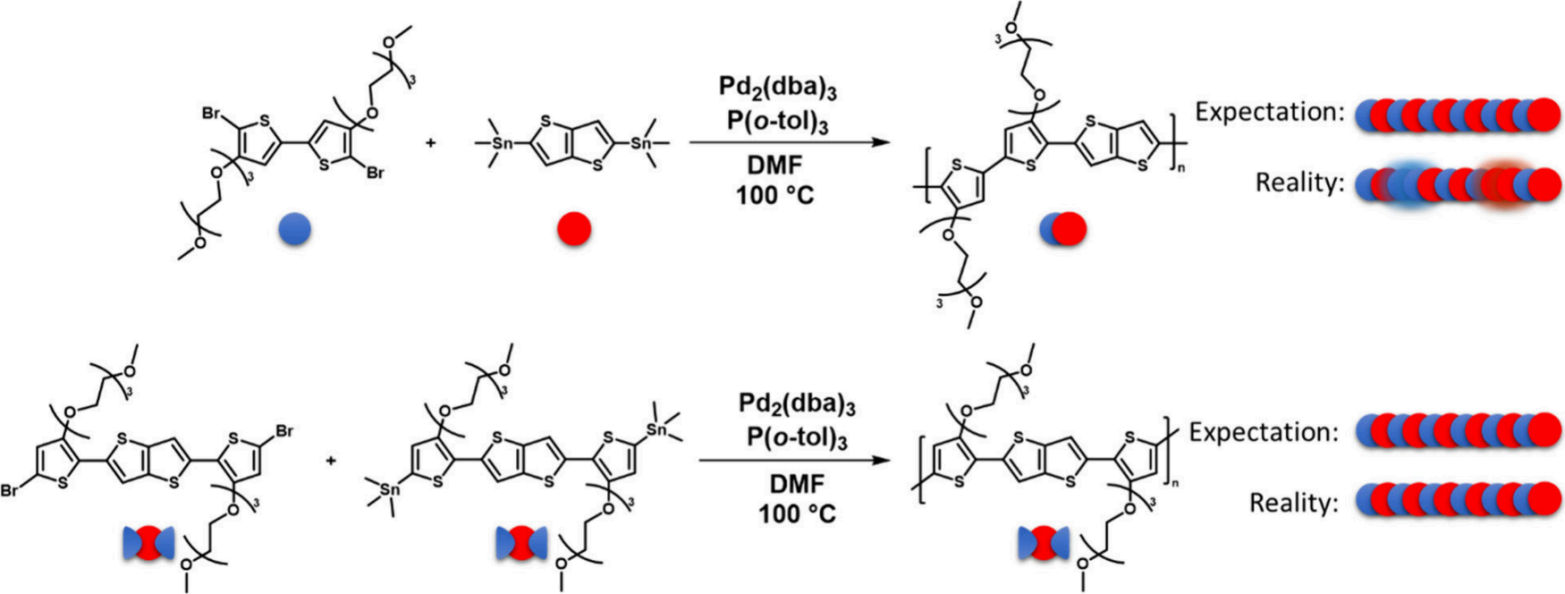
Synthesis of Conventional and Homocoupling-Free pgBTTT^a^

a Top: Reported synthesis route for pgBTTT,^33^ employing a standard Stille polymerization, with a schematic representation of the homocoupling sequences that are introduced. Bottom: Symmetric Stille polymerization for homocoupling-free pgBTTT.

To elucidate the impact of homocoupling defects in a benchmark OECT material, pgBTTT was synthesized both via the standard Stille polymerization and an alternative symmetric Stille route (Scheme 1, Scheme S4). In the symmetric monomer polymerization, any self-coupling between brominated or stannylated monomers still yields a perfectly alternating gBT-TT backbone, thereby eliminating homocoupling defects. Matrix-assisted laser desorption/ionization – time-of-flight mass spectrometry (MALDI-ToF MS) and electrospray deposition – scanning tunneling microscopy (ESD-STM) were used to establish the structural integrity and the molar mass distributions of the polymers. We assess the influence of homocoupling defects on device performance by comparing the performance of both OMIECs in OECTs. It was found that the symmetric polymerization yields a polymer with a significantly increased performance (μC*) compared to conventionally synthesized pgBTTT.

The symmetric Stille polymerization was found to completely avoid homocoupling while simultaneously yielding polymers of higher molar mass. Thus, this approach provides structurally well-defined materials, while also offering access to previously unachieved molar masses under identical reaction conditions. We therefore expect that it will contribute to more reproducible devices and more meaningful comparison with theoretical models. Together, these advances allow a more rigorous evaluation of how molecular precision and macromolecular properties govern charge transport in high-performance p-type OECTs.

## Results and Discussion

Conventional pgBTTT was synthesized according to a literature procedure (see Supporting Information, SI).^33^ For the synthesis of homocoupling-free pgBTTT, different methods were explored. Ultimately, a symmetric, extended monomer was designed consisting of a thieno­[3,2-*b*]­thiophene core flanked by glycolated thiophenes, to avoid the possibility of TT and gBT homocouplings. While symmetric polymerization has been applied before using oxidative conditions,^11,40–42^ this was scarcely successful here with glycolated monomers, and substantial synthetic optimization was hence required, as detailed in the SI (Section 2.3). It was found that symmetric polymerization via Stille affords the best results, namely a high molar mass and homocoupling-free batch of pgBTTT (as obtained from the chloroform Soxhlet fraction). For this synthetic route, one portion of the symmetric monomer was brominated, while the other part was stannylated. Subsequent Stille polymerization can therefore only result in the target structure (Scheme 1), regardless of whether homocoupling occurs or not. Details of the monomer and polymer syntheses can be found in the SI. ^1^H NMR spectra of the two polymers (Figure S4) look very similar, illustrating that the sensitivity of NMR is not sufficient to assess subtle structural differences, in particular under the employed experimental conditions (CDCl_3_, room temperature), for which aggregation of the pgBTTT chains further broadens spectral features.

UV–vis-NIR absorption spectroscopy was conducted on films of conventional and homocoupling-free pgBTTT, revealing that homocoupling sequences do not significantly affect the absorption spectrum (Figure S15). To probe some of the electrochemical properties, cyclic voltammetry measurements were performed. The absence of homocoupling does not appear to have a notable impact on the frontier molecular orbital energy levels (HOMO and LUMO) or on the oxidation onsets (Table S2). The aqueous voltammograms (Figure S16, S17) are similar to each other and to the originally reported result for pgBTTT.^33^

To gain deeper insights into the polymer structures, MALDI-ToF MS analysis was performed (Figure 1). As expected, conventional pgBTTT displays numerous signals representing differing gBT:TT ratios. On the contrary, pgBTTT prepared by the alternative symmetric approach consists of only chains exhibiting the desired ratio. While this is a strong indication of the significant difference in homocoupling content between the two pgBTTT batches, MALDI-ToF MS does not allow a straightforward quantification of the defect ratio. ESD-STM was hence employed to verify this observation in a quantitative manner and ascertain the nature of these homocoupling defects.^11,43–47^ Figure 2 compares the chloroform (Soxhlet) fractions of the symmetric (a) and conventional (b) Stille polymers as imaged by ESD-STM. For both polymers, highly resolved STM images clearly show linear backbones and side chains interdigitating with a different degree of overlap between them. The images were carefully analyzed and fitted with molecular dynamics optimized molecular models of the different comonomers to identify potential homocoupling defects in the backbones.^11,34,43–46^ To account for the different synthesis protocols, models of the gT-TT-gT unit were used to fit homocoupling-free pgBTTT, while separated models for gBT and TT were employed for conventional pgBTTT. Importantly, no defects were detected in the symmetric Stille polymer, whereas over 9% of the monomer couplings in the conventional Stille batch were homocoupling defects. Statistics on the different types of homocoupling defects identified in this batch are presented in Figure 2c.

**Figure 1:**
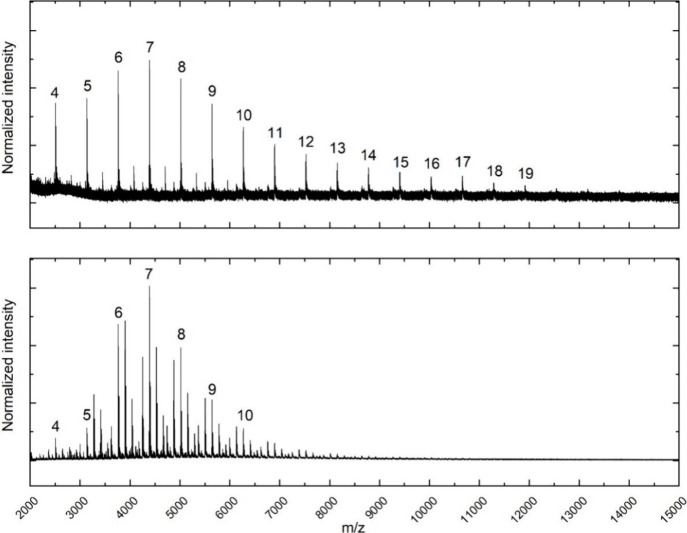
MALDI-ToF mass spectra (*m*/*z* range from 2 to 15 kDa) for homocoupling-free pgBTTT (top) and conventional pgBTTT (bottom). The chains containing a 1:1 ratio of gBT:TT units are indicated with their number (*n*) of ‘gBTTT’ repeat units above the respective peaks.

**Figure 2:**
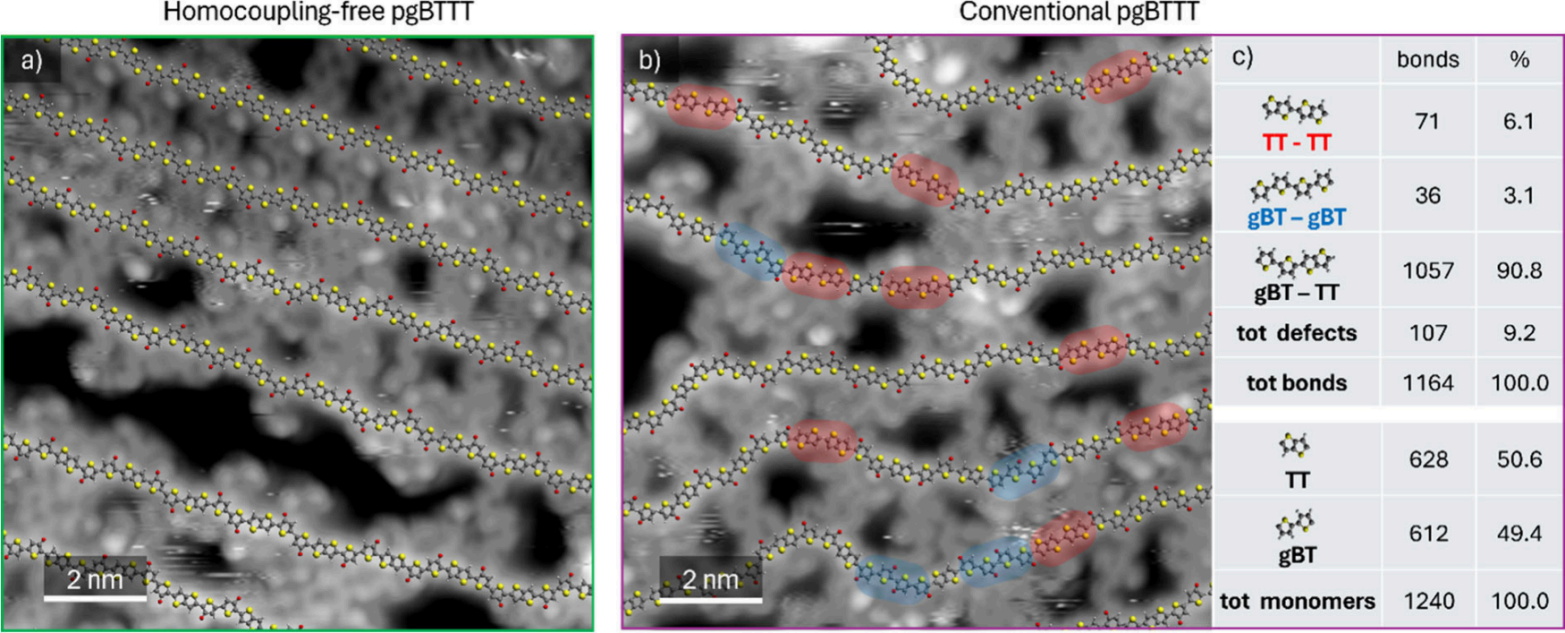
Examples of STM images of pgBTTT on a Au(111) surface, synthesized by a) symmetric Stille and b) standard Stille polymerization. Molecular models of the backbones are overlapped onto the experimental images and the different types of homocoupling defects (double thienothiophene, TT, or double glycolated bithiophene, gBT) are highlighted in red and blue, respectively. The defect frequencies observed in conventional pgBTTT are shown in c).

Another striking difference between the two polymers in the MALDI-ToF MS spectra (Figure 1) is that homocoupling-free pgBTTT shows chains of higher molar mass. Thus, from the MS data it seems that the symmetric Stille polymerization delivers a polymer containing only chains with the desired 1:1 ratio of gBT and TT units and with a higher molar mass, using the same reaction conditions as applied for the standard Stille polymerization. Attempts to confirm the molar mass difference between the two polymers using gel permeation chromatography (GPC) in chloroform, chlorobenzene, or *N*,*N*-dimethylformamide were unsuccessful because the obtained GPC traces were either too broad or very low in intensity. This issue, caused by polymer aggregation or solubility issues in common GPC solvents, has been previously reported for glycolated polymers and frequently leads to an overestimation of the molar masses of these materials.^48–50^ Recently, several studies have omitted GPC for the molar mass determination of such polymers, using alternative techniques like MALDI-ToF MS instead.^24,31^ However, as previously noted, MALDI-ToF MS is not quantitative and can indicate molar mass trends but does not provide precise values.^51^ Currently, the only technique that accurately measures the exact number- or weight-average molar masses (*M*_n_ and *M*_w_, respectively) and polymerization degrees (DP_n_) of glycolated OMIECs, while also attaining the full mass distribution, is ESD-STM.^44^

Measuring the length of line profiles traced along the polymer backbones in STM images for a large number of polymers enables the collection of complete mass distributions.^44^ Figure 3 shows representative STM images of low-coverage areas of homocoupling-free and conventional pgBTTT, where individual polymer profiles are clearly distinguishable. The corresponding STM-derived mass distributions, with relevant mass numbers, are displayed in Figure 3c,d. A detailed description of the length-profile acquisition and the process to obtain the mass distributions is provided in the SI (Section 6). The results clearly show a significant difference in the lengths of the polymers for the two batches, with homocoupling-free pgBTTT chains being (on average) significantly longer than the conventional Stille ones, quantitatively confirming the observations derived from MALDI-ToF MS.

**Figure 3:**
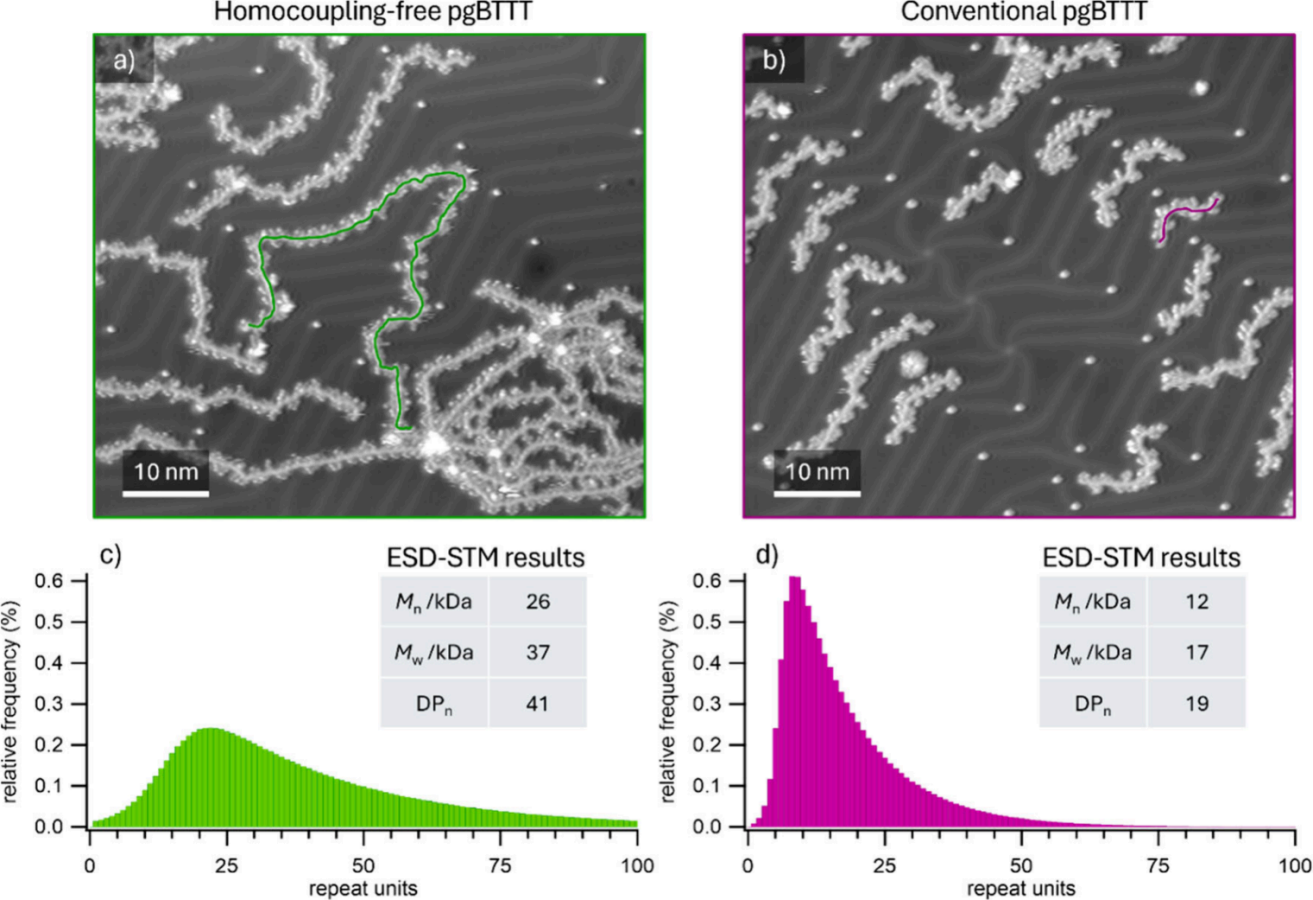
Examples of typical larger-scale STM images of a) symmetric Stille pgBTTT and b) conventional pgBTTT, used to construct the mass distributions. The lengths of the exemplary polymer profiles highlighted by colored lines in each image are 78 nm in a) and 11 nm in b), respectively. Experimental ESD-STM mass distributions (corrected by survival analysis, see SI) of c) symmetric Stille pgBTTT and d) conventional pgBTTT, alongside the values obtained for the statistical parameters *M*_n_, *M*_w_, and DP_n_ (using the same gBTTT repeat unit for both polymers).

The higher frequency of homocouplings and the lower mass observed in conventional pgBTTT, compared to homocoupling-free pgBTTT, could suggest that such defects inhibit polymer chain growth. To investigate whether the presence of homocoupling defects indeed affects, and in particular hinders, the growth of polymer chains in conventional pgBTTT, we examined whether defects are uniformly distributed along the polymer chains and whether shorter polymers exhibit a higher number of homocouplings. The precise polymer sequencing enabled by ESD-STM allowed us to conduct three statistical tests to assess potential correlations between: (i) consecutive defects along the backbone, (ii) defect density and polymer length, and (iii) defective bonds and polymer ends. Our detailed analysis (see SI) revealed no statistically significant correlations in any of these tests. These observations indicate that homocoupling defects do not locally suppress polymer growth through chain-specific or position-dependent mechanisms. While it should be noted that the samples analyzed by ESD-STM were not crude products directly obtained from the polymerization reaction but rather those subjected to Soxhlet extractions, it is difficult to envision how the Soxhlet extractions could have selectively removed polymer chains exhibiting such correlations. Within this context, our findings suggest that homocoupling defects are unlikely to play a primary role in limiting polymer growth during Stille polymerization through local, defect-induced mechanisms, thereby challenging commonly held assumptions.

At the same time, a distinct global effect of homocoupling defects must be considered. Our measurements indicate that the TT and the gBT homocoupling reactions occur with different frequencies (Figure 2), suggesting that one monomer type is consumed more rapidly than the other during polymerization. This generates an effective stoichiometric imbalance as the reaction proceeds, which can reduce the degree of polymerization, in a manner analogous to that described by the Carothers equation for systems with genuine stoichiometric imbalance. A quantitative evaluation of this effect is nontrivial but simple estimates based on generalizations of Carothers equation suggest that the observed difference in homocoupling frequencies (∼3%) is unlikely to account for the nearly 2-fold reduction in the DP_n_ observed experimentally (Figure 3). It should finally be noted that an additional Soxhlet solvent (dichloromethane, DCM) was applied for homocoupling-free pgBTTT, which aids in removing shorter polymer chains (SI, Section 2.3).

The impact of homocoupling defects on device performance was assessed by incorporating the polymers in OECT channels with various aspect ratios (Table 1, Figure 4, S22, S23). All transfer curves were measured in the linear regime to ensure stable operation at low charge carrier densities.^31^ The product of the electronic mobility and volumetric capacitance (μC*) was extracted from the transfer curves and used as the figure of merit. Since the ESD-STM data revealed a distinct molar mass difference, and higher molar mass is generally associated with improved OECT performance,^38,39^ this factor has to be taken into account. When comparing the highest molar mass (chloroform) fractions of both conventional and homocoupling-free pgBTTT, the latter exhibited significantly better performance (μC* = 1020 ± 80 F cm^–1^ V^–1^ s^–1^ vs 1940 ± 190 F cm^–1^ V^–1^ s^–1^). To distinguish the effects of molar mass and structural defects, the lower molar mass (DCM) Soxhlet fraction of homocoupling-free pgBTTT was included in the comparison. MALDI-ToF MS analysis suggests that the DCM fraction of homocoupling-free pgBTTT has a molar mass similar to the chloroform fraction of conventional pgBTTT (compare Figure S7 and S14), making them more comparable as OECT channel materials. An intermediate μC* of 1390 ± 80 F cm^–1^ V^–1^ s^–1^ was observed for this DCM fraction, indicating that when polymers of similar molar mass are compared, the homocoupling-free polymer still outperforms conventional pgBTTT. These findings imply that the removal of homocoupling defects contributes to enhanced OECT performance, but that the increased molar mass, accessed through the symmetric polymerization route, is the predominant factor governing the observed improvement.^35,38,52–55^ The combined effects of removing homocoupling and increasing molar mass amount to a total μC* enhancement of 89%. All three materials show comparable operational stability during cycling measurements (100 cycles), with changes in *I*_D_ and *g*_m_ on the order of ± 10% over the cycling period for all devices (Figure S25).

**Figure 4:**
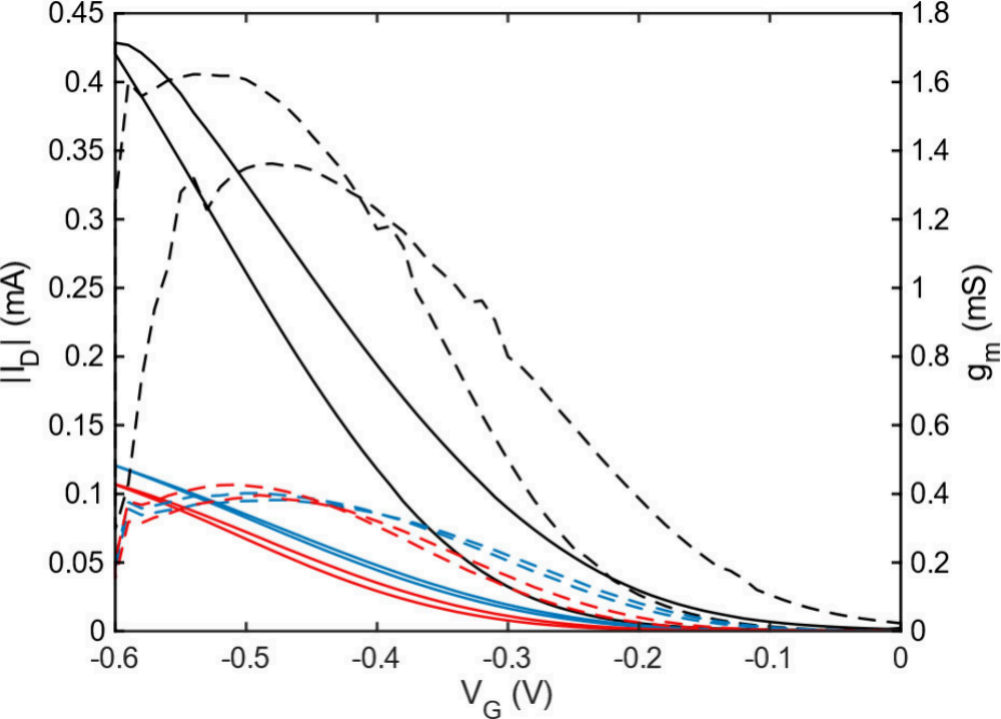
Transfer curves (*I*_D_ vs *V*_G_ with *V*_D_ = −0.1 V, solid lines) and voltage dependent transconductance (*g*_m_, dashed lines) for OECT devices prepared from homocoupling-free pgBTTT (black), conventional pgBTTT (blue), and lower molar mass homocoupling-free pgBTTT (DCM fraction) (red), with channel dimensions *W* = 100 μm and *L* = 200 μm.

**Table 1:**
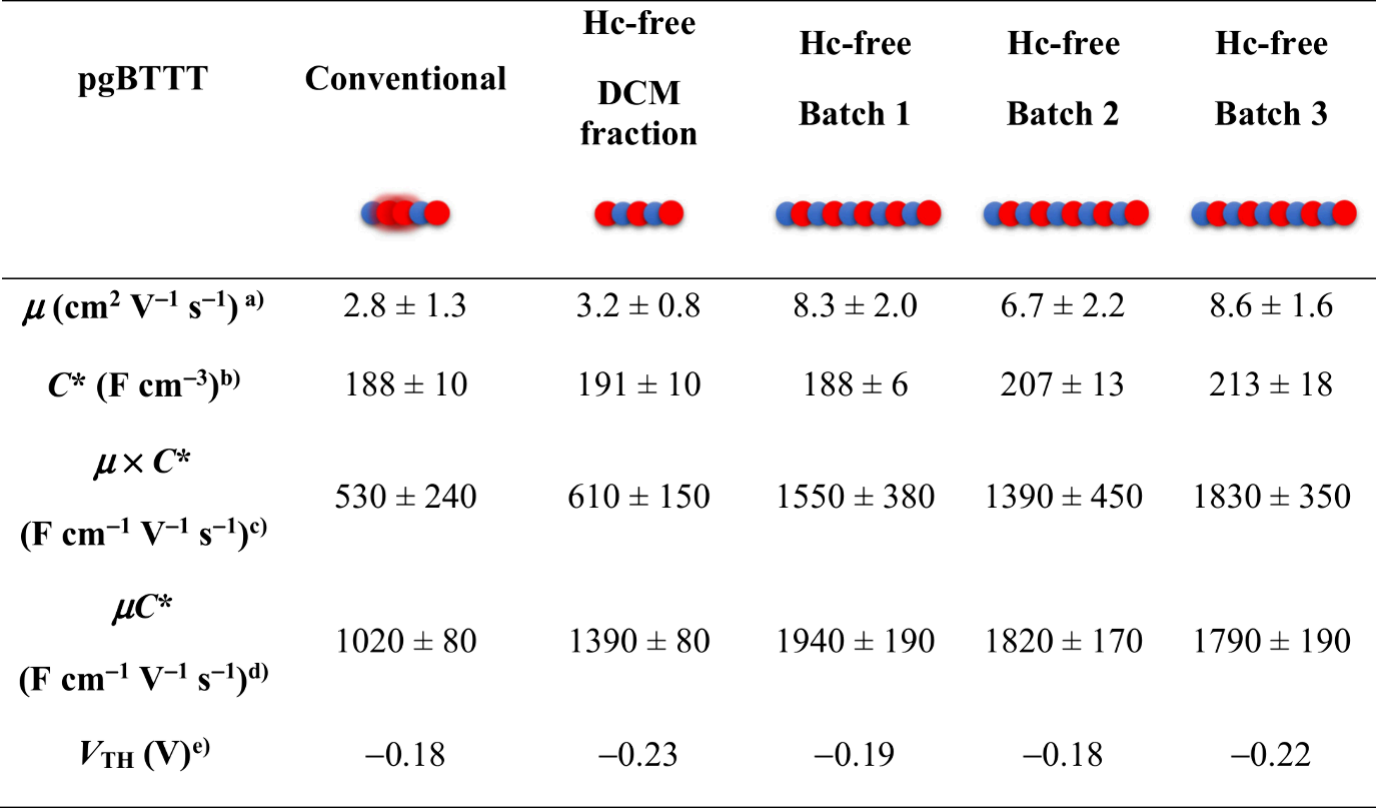
Summary of OECT Performance Parameters for Conventional pgBTTT (Chloroform Fraction) and Homocoupling-Free (hc-Free) pgBTTT^a^

a Calculated by measuring the electronic charge carrier transit time while pulsing constant gate current. The data were collected using devices with *L* = 500, 200, and 100 μm. Reported uncertainties are one standard deviation, with *n* = 5 devices. An approximate 3-fold increase in μ is observed for homocoupling-free pgBTTT compared to conventional pgBTTT, calculated based on the average μ from batch 1, 2, and 3.

b Independently determined by EIS. Reported uncertainties are one standard deviation, with *n* = 3–5 devices.

c Calculated by multiplying the independently determined μ and C* values. Reported uncertainties are one standard deviation.

d Extracted from the relationship between the transconductance values measured in the linear regime and the operating parameters (drain voltages and channel dimensions) by linear regression. The coefficient of determination, *R* ^2^, was larger than 0.8 for all batches. For each material, the RMS error is reported, calculated by performing a linear regression, with *n* = 15 samples.

e Extracted in the saturation regime (*V*_D_ = −0.5 V) of each device (*n* = 5) by fitting the *I*_D_^1/2^ vs *V*_G_ plots.

f Three independent batches of hc-free pgBTTT (chloroform fraction) were evaluated to demonstrate the reproducibility of the symmetric polymerization. The lower-molar-mass DCM fraction of hc-free pgBTTT (batch 1) was additionally measured to disentangle the effects of molar mass and homocoupling defects.

It is important to note here that both conventional and homocoupling-free pgBTTT were synthesized under identical reaction conditions, demonstrating the superiority of the symmetric Stille polymerization in producing homocoupling-free polymers with high molar mass. To further confirm the robustness of this method, its reproducibility was tested by synthesizing two additional batches of homocoupling-free pgBTTT, which were also evaluated in OECTs (Table 1). In both cases, similar μC* values were achieved, reinforcing the reliability of this new approach in producing high-performance OECT channel materials.

To determine if the enhanced performance originates from an improved electronic (hole) transport (μ) or from an increased uptake of ions (C*), both figures of merit were also measured independently. Electrochemical impedance spectroscopy (EIS) was used to determine C* at doping potentials of 0.4 and 0.5 V (Figure S27–S31). All polymers exhibit similar C* values around 200 F cm^**‑**3^. The observed differences in the μC* product can thus mainly be attributed to an increase of the charge carrier mobility for homocoupling-free pgBTTT. This was confirmed by measuring the electronic charge carrier transit time while pulsing constant gate current to extract the μ values (Figure S26, Table S6). It was found that the combination of a perfectly alternating backbone structure and increased molar mass amounts to an average 3-fold enhancement of μ for pgBTTT.^38,39,56^

By measuring μ and C* separately, a comparison could be made between μC*, derived from the OECT transfer curve, and μ **×** C*, calculated by multiplying the independently measured values (Table 1, Figure S23). As shown in Table 1, a significant discrepancy was observed between the absolute values of these metrics for conventional pgBTTT and for the DCM fraction of hc-free pgBTTT. Notably, only in high molar mass batches of hc-free pgBTTT, μC* and μ **×** C* yielded values in reasonable agreement. Shahi et al. highlighted before that transfer curve-derived μC* values can deviate from the actual device performance.^57^ The strong agreement between μC* and μ **×** C* for high molar mass, homocoupling-free polymers suggests that these materials yield more reliable device properties, accurately reflecting their intrinsic OECT performance characteristics. Compared to similar thiophene-based p-type channel materials operating in enhancement mode, and measured in devices of comparable dimensions, homocoupling-free pgBTTT demonstrates a record performance (Figure S24).^17,29,33,58–62^ This shows that our polymerization strategy effectively unlocks previously untapped potential in these types of materials.^63^

To gain insights in the solid-state microstructure of conventional pgBTTT and the homocoupling-free polymer, 2D grazing-incidence wide-angle X-ray scattering (GIWAXS) analysis was performed on films of both materials. Based on the scattering patterns (Figure 5a,b and S32), there is some variation in crystalline texture: whereas conventional pgBTTT is predominantly edge-on, the homocoupling-free variant contains a larger population of face-on crystallites in both the high and low molar mass cases. An overview of all the observed signals can be found in Table S7. When looking at the lineouts in Figure 5c,d, two orders of lamellar stacking (*h*00, *Q*_*z*_ = 0.42 and 0.86 Å^–1^) are clearly present in both materials, which could indicate (relatively) long-range order of the crystallites.^33^ The lamellar stacking distance is in good agreement with literature, amounting to 14.97 Å, and is the same for both the conventional and homocoupling-free material. Coherence length estimates (Table S8), calculated via the Scherrer equation on the (100) lamellar stacking peak, indicate a significantly smaller coherence length for the homocoupling-free material, possibly indicative of smaller crystallites. Clearly visible in the *Q*_*z*_ lineout is the larger intensity of the (010) π-π stacking peak in the homocoupling-free case (*Q*_*z*_ = 1.72 Å^–1^, 3.70 Å), indicating a larger portion of face-on orientation. The lower molar mass (DCM) fraction of the homocoupling-free material exhibits tighter packing (*Q*_*z*_ = 1.77 Å^–1^, 3.55 Å), while conventional pgBTTT (*Q*_*z*_ = 1.75 Å^–1^, 3.59 Å) shows an intermediate value. This is similarly observed for the (010) peaks in the *Q*_*xy*_ direction and seems to be molar mass dependent, with lower molar mass pgBTTT showing tighter π-π stacks. The backbone seems to be more tilted relative to the substrate in the homocoupling-free material, as evidenced by the signal appearing stronger at ≈25° relative to the *Q*_*xy*_ axis (Figure 5b). In general, the absence of homocoupling defects results in a mixed edge-on and face-on morphology and large anisotropy in the film. Together with the higher molar mass, this might allow for the occurrence of more tie-chains and stronger percolation pathways for the charges. Given the bulk conductive properties of OMIECs, this suggests a stronger 3D entangled network of polymer chains in the homocoupling-free material, which may benefit mobility.^63–65^

**Figure 5:**
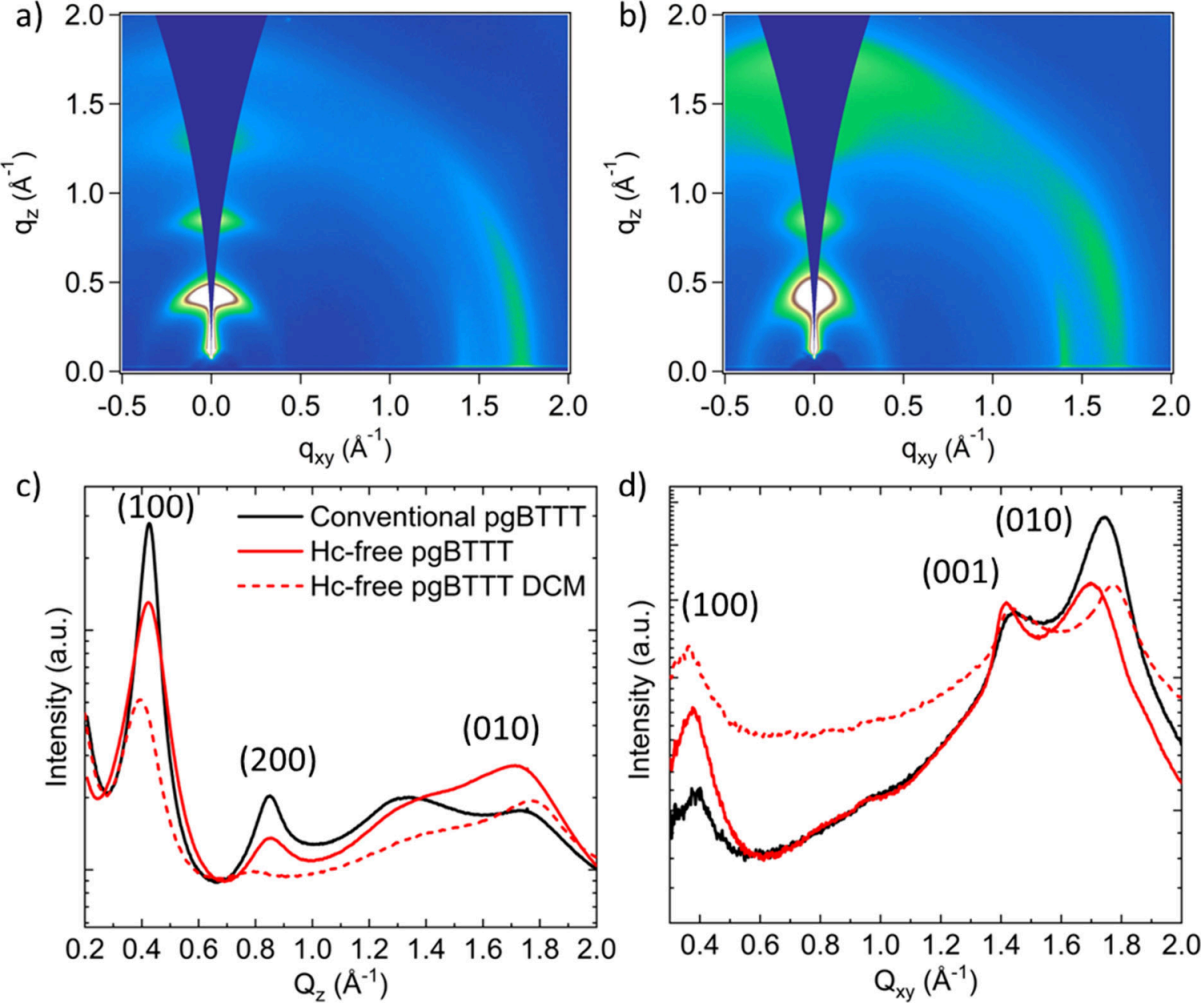
Two-dimensional GIWAXS patterns for a) conventional and b) (high molar mass) homocoupling-free pgBTTT. c) In-plane (*Q*_*z*_) and d) out-of-plane (*Q*_*xy*_) thickness-normalized lineouts for conventional and homocoupling-free pgBTTT. Dashed and continuous lines indicate the medium molar mass dichloromethane and the high molar mass chloroform fraction, respectively.

## Conclusions

In this study, we investigated the influence of homocoupling defects and molar mass on the performance of the benchmark OECT polymer, pgBTTT. By employing a symmetric Stille polymerization, homocoupling-free polymer chains with significantly higher molar mass were obtained under identical reaction conditions. The absence of structural defects and the enhanced chain length were confirmed by complementary MALDI-ToF MS and ESD-STM analysis. Comparative OECT evaluations revealed that while the removal of homocoupling defects in itself already results in an improvement in device performance, the increase in molar mass is the main factor causing the significantly enhanced μC* product. These findings highlight the importance of controlling both molecular precision and macromolecular properties to achieve reproducible and high-performing OECT materials.

Interestingly, detailed statistical analysis of STM images revealed no significant correlation between homocoupling defects and polymer growth, challenging the long-standing assumption that such defects inherently limit chain elongation. This assumption was likely prevalent due to the prior unavailability of synthetic methods capable of producing fully homocoupling-free samples, coupled with the lack of precise analytical techniques to reliably quantify defect ratios.

Our work underscores the importance of homocoupling-free synthesis, not only for ensuring structural fidelity and reproducibility, but also for enabling a clear understanding of structure–property relationships in polymeric OMIECs. While demonstrated here for a p-type system, the same design principles are also applicable to symmetric n-type materials, where defect control may be even more critical given their currently lower device performance. We therefore encourage our peers to adopt similarly well-controlled polymerization strategies to uncover the intrinsic potential of emerging OMIECs and to accelerate their translation into practical OECT applications and beyond.

## Supplementary Material



## Notes

General methods section, synthesis of conventional and hc-free pgBTTT (including optimization efforts for the latter), ^1^H and ^13^C NMR spectra, MALDI-ToF mass spectra, cyclic voltammetry measurements, ESD-STM analysis, ICP-MS measurements, OECT, mobility and EIS measurements, and GIWAXS analysis, including Schemes S1–S4, Figures S1–S32, and Tables S1–S8 (PDF)
