# Supplementary material for: Revealing the Full Potential of Glycolated Mixed Ionic-Electronic Semiconductors – Symmetric Monomer Polymerization to Boost Electrochemical Transistor Performance
Source: J Am Chem Soc. 2026 Feb 23;148(8):8383–92. doi: 10.1021/jacs.5c19024 (PMC12964417; doi:10.1021/jacs.5c19024)
Supplement: Supplementary file 1 [file ja5c19024_si_001.pdf]

Revealing the Full Potential of Glycolated Mixed Ionic-Electronic Semiconductors – Symmetric Monomer Polymerization to Boost Electrochemical Transistor Performance

Lize Bynens,[†,‡,♦] *Paola Mantegazza,*[§,♦] *Adam Marks,*[¶] *Yeongmin Park,*[¶] *Arwin Goossens,*[#,‡] *Stefania Moro,*[§] *Tyler J. Quill,*[¶] *Garrett Lecroy,*[¶] *Christina Cheng,*[¶] *Arianna Magni,*[¶] *Laurence Lutsen,*[†,‡] *Jochen Vanderspikken,*[*,†,‡] *Simon E. F. Spencer,*[°] *Koen Vandewal,*[#,‡] *Alberto Salleo,*[¶] *Giovanni Costantini,*[*,§,±] *Wouter Maes*[*,†,‡]

[†] Hasselt University, Institute for Materials Research (imo-imomec), Design & Synthesis of Organic Semiconductors (DSOS), Martelarenlaan 42, B-3500 Hasselt, Belgium

[‡] imec, imo-imomec, Wetenschapspark 1, B-3590 Diepenbeek, Belgium

[§] School of Chemistry, University of Birmingham, Edgbaston, Birmingham, B15 2TT, United Kingdom

[¶] Department of Materials Science and Engineering, Stanford University, Stanford, California 94305, United States

[#] Hasselt University, Institute for Materials Research (imo-imomec), Organic Opto-Electronics (OOE), Martelarenlaan 42, B-3500 Hasselt, Belgium

[°] Department of Statistics, University of Warwick, Coventry CV4 7AL, United Kingdom

[±] School of Physics and Astronomy, University of Birmingham, Edgbaston, Birmingham, B15 2TT, United Kingdom

[♦] These authors contributed equally to this work.

Table of contents

1. General methods S3
2. Materials synthesis S6
    2.1. Synthesis of conventional pgBTTT S6
    2.2. Synthesis of homocoupling-free pgBTTT S7
    2.3. Optimization efforts toward homocoupling-free pgBTTT S14
3. MALDI-ToF MS S18
4. UV-Vis-NIR absorption spectroscopy S22
5. Cyclic voltammetry S23
6. ESD-STM S24
    6.1. Analysis of defects and their correlations in polymer sequences S24
        6.1.1. Analysis of the correlation between consecutive defects S24
        6.1.2. Analysis of the density of defects as a function of polymer length S26
        6.1.3. Analysis of the density of defects at the polymer ends S28
    6.2. Polymer 2D assembly and side chain interdigitation patterns S30
    6.3. Analysis of the mass distributions S32
7. ICP-MS S35
8. OECTs S36
9. Mobility measurements S40
10. Electrochemical impedance spectroscopy S42
11. GIWAXS S45
12. References S47

1. General methods

Materials and characterization: All reagents and chemicals were purchased from commercial sources and used without further purification. A solvent purification system (MBraun, MB-SPS-800) equipped with alumina columns was used to obtain dry solvents. Preparative (recycling) gel permeation chromatography was performed on a JAI LaboAce LC-7080 Plus system equipped with JAIGEL 1H and 2H columns (eluent: $CHCl_3$, flow rate: 10 mL min^{-1}). NMR spectra were recorded on a Jeol NMR spectrometer operating at 400 MHz for ^{1}H and at 100 MHz for ^{13}C. All chemical shifts (δ, in ppm) were established relative to $CDCl_3$ (δ = 7.26 ppm for ^{1}H NMR, δ = 77.16 ppm for ^{13}C NMR). A Bruker UltrafleXtremeTM MALDI-ToF/ToF system was used to record the MALDI-ToF mass spectra of the polymers and monomers. Sample preparation consisted of mixing 10 µL of the matrix solution (25 mg mL^{-1} *trans*-2-[3-(4-*tert*-butylphenyl)-2-methyl-2-propenylidene]malononitrile (DCTB) in chloroform) with 3 µL of the analyte solution (5 mg mL^{-1} in chloroform), after which 0.5 µL of the mixture was spotted onto an MTP Anchorchip 600/384 MALDI plate. Ultraviolet-visible-near-infrared (UV-Vis-NIR) absorption spectra were recorded (and background corrected) on a Cary 5000 UV-Vis-NIR spectrophotometer (Agilent) using a spectral band width of 2 nm. The films for the UV-Vis-NIR absorption measurements were prepared by spin-coating (for 1 min at 1000 rpm) 100 µL of a polymer solution (6 mg mL^{-1}) in $CHCl_3$ on a glass slide after heating the solution at 40 °C for 20 h. Cyclic voltammograms were recorded using an Autolab PGSTAT30 from Methrohm controlled with GPES software (version 4.9). Cyclic voltammetry (CV) in an organic solvent was carried out in argon-purged acetonitrile at room temperature with a 0.1 M tetrabutylammonium hexafluorophosphate ($TBAPF_6$) electrolyte. A one-compartment microcell designed for three-electrode configuration was fitted with a Pt wire working and counter electrode, and an $Ag/AgNO_3$ reference electrode (Ag wire immersed in electrolyte containing 0.01 M $AgNO_3$). A film of the polymer analyte was formed on the working electrode by casting it from a chloroform solution. Voltammograms were recorded at a scan rate of 100 mV s^{-1} under a constant argon flow. Typically, 5 scans per film were recorded and the highest occupied and lowest unoccupied molecular orbital energy levels were estimated from the averaged onset potentials of the 3rd, 4th, and 5th scan. The onset potential was determined from the intersection of two tangents drawn at the rising and background current of the voltammogram. Ferrocene was used as an external standard. For aqueous CV, a standard three-electrode configuration featuring an Ag/AgCl reference electrode, a Pt counter electrode, and a polymer-coated ITO glass slide as the working electrode were used. The ITO glass slides

were cleaned by sonication in acetone, methanol, and isopropanol, after which the polymers were deposited by spin-coating (for 1 min at 1000 rpm) 100 µL of 6 mg mL^{-1} solutions (after overnight heating at 40 °C). The CV measurements were carried out at 100 mV s^{-1} in an aqueous electrolyte consisting of a 0.1 M NaCl in distilled water, which was degassed with an argon flow for 10 min prior to the measurement. Typically, 6 scans per film were recorded and the oxidation onsets were determined from the 6th scan with the intersection of the baseline and the tangent at the point of the highest slope.

ESD-STM: The STM images were acquired using a low temperature scanning tunneling microscope (LT-STM) (CreaTec Fischer & Co. GmbH) equipped with a bath cryostat. All images were acquired in constant current feedback mode at a temperature of –196 °C. The samples were prepared by dissolving pgBTTT in chlorobenzene at a concentration of ≈0.025 g L^{-1} and adding methanol to a obtain a 4:1 chlorobenzene:methanol volume ratio. The polymers were deposited from solution by electrospray deposition (ESD) with a 4-stage Molecular Spray system. The deposition current was monitored on the target substrate and the deposition charges ranged from 6 pAh to 10 pAh. The (111) orientation of a gold single crystal (Surface Preparation Laboratory) was used as substrate and prepared in ultrahigh vacuum (UHV) by cycles of argon ion sputtering (1 kV) and annealing to 500 °C. The STM images were processed with the WSxM[1] and Gwyddion[2] software, while the molecular models were made and optimized in Avogadro.[3] Fitting of the STM images with these molecular models was performed with the LMAPper software.[4]

GIWAXS: Samples were spin-coated at 1000 rpm onto a native oxide silicon substrate from a 10 mg mL^{-1} solution in $CHCl_3$. GIWAXS measurements were performed at the Stanford Synchrotron Radiation Lightsource on beamline 11-3, with an X-ray energy of 12.7 keV and an incidence angle of 0.1°. The sample to detector distance was 309.1 mm and calibrated to a polycrystalline LaB6 standard. Measurements were performed in a helium chamber to minimize air scattering. All data were corrected for the geometric distortion of the flat detector used, normalized by exposure time, sample thickness, and monitor counts, and analyzed using Nika 1D SAXS1[5] and WAXstools[6] software in Igor Pro.[5] Lineouts were taken at azimuthal angle ranges of −25 to +25° and 70 to 88° for Q_z and Q_{xy} lineouts.

OECTs: OECT devices were fabricated using lithographically patterned chips with gold contacts and two parylene layers; one to passivate the gold lines on the chip and the other as the sacrificial layer to confine the polymer channels. The chips were gently rinsed with acetone and isopropanol and then treated with UV-ozone for 10 min. pgBTTT was spun at 1000 rpm for 60 s and the film was annealed at 60 °C for 60 s. The channel was patterned by lifting off

the parylene layer, leaving the pgBTTT film with the designed channel dimensions (width of 100 μm and different lengths of 10, 50, 100, 200, and 500 μm). Channel thicknesses were measured with a Bruker Dektak profilometer. For each chip, the thickness was averaged over 5 points, resulting in 69 nm for conventional pgBTTT, 38 nm for homocoupling-free pgBTTT (DCM fraction), and 62 nm, 107 nm, and 91 nm for homocoupling-free pgBTTT batch 1, 2, and 3, respectively. All OECT measurements were performed using a solution of 100 mM NaCl in deionized water as the electrolyte and an Ag/AgCl pellet (Warner Instruments) as the gate electrode. The aqueous electrolyte solution was degassed by bubbling argon gas for 30 min and then drop-cast on the devices. Transfer and output curves were measured using a Keithley 2612 Source Meter and custom LabVIEW software in a nitrogen-purged glove box. The devices were operated at drain voltages (V_D) of 10, 50, and 100 mV while sweeping the gate voltage (V_G) from 0 to −0.6 V with a scan rate of 100 mV s^{-1}. Stability measurements were performed on OECTs with channel dimensions of 50 μm in length and 100 μm in width. Each OECT device was cycled 100 times between $V_G = 0$ and $V_G = -0.6$ V at a $V_D = -0.1$ V with a scan rate of 100 mV s^{-1}.

Electrochemical impedance spectroscopy: EIS was performed for determination of the capacitance using a PalmSens Sensit Smart. The impedance spectra were measured in 0.1 M NaCl aqueous electrolyte with a polymer-coated OECT channel (width of 6 mm and total lengths of 5, 4, 3, and 2.5 mm) used as the working electrode (20 nm Cr and 80 nm Au source and drain electrodes shorted), an Ag/AgCl pellet (A-M Systems) as the reference electrode, and a Pt wire (Thermo Fisher Scientific) as the counter electrode. The measurement was realized over a frequency range of 10^5 to 0.1 Hz with a 10 mV AC amplitude and at DC offset potentials of 0.4 and 0.5 V. Premeasurement treatment at the DC offset potential during 30 s was employed. The impedance spectra were fit to an equivalent RC circuit, where R represents the resistance of the electrolyte and C the capacitance of the semiconducting polymer film, using the *impedance.py* Python package.[7] The extracted capacitance values were normalized by the film volume to obtain the volumetric capacitance (C*). Film thickness was measured with a Bruker DektakXT profilometer before exposure to the electrolyte.

2. Materials synthesis

2.1. Synthesis of conventional pgBTTT

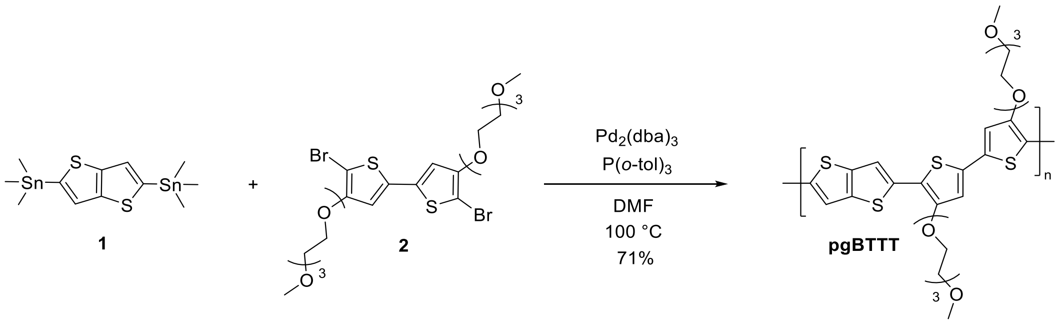

Scheme S1. Synthesis of conventional pgBTTT via the standard Stille polymerization.[8]

pgBTTT[8]: The two monomers were prepared according to literature.[8,9] The polymerization reaction was carried out following an adapted literature procedure.[8] A schlenk tube was charged with compound **1** (292 mg, 0.627 mmol, 1 eq.), bithiophene **2** (407 mg, 0.627 mmol, 1 eq.), tris(dibenzylide-neacetone)dipalladium(0) (11.5 mg, 12.5 µmol, 0.02 eq.), tri(*o*-tolyl)phosphine (15.3 mg, 50.2 µmol, 0.08 eq.), and 10 mL of anhydrous degassed DMF. The schlenk tube was then brought under inert atmosphere (using 5 vacuum/Ar cycles) and heated overnight at 100 °C. Afterwards, the mixture was diluted with DMF, the Pd scavenger diethylammonium diethyldithiocarbamate was added, and the mixture was stirred for 1 h at 100 °C. Subsequently, the reaction mixture was added to 100 mL of cold methanol. The polymer precipitate was subjected to Soxhlet extractions with methanol, acetone, hexanes, tetrahydrofuran (THF), and chloroform. The chloroform fraction was evaporated to a minimum volume and once again added to methanol. The precipitate was filtered off and dried under high vacuum. This resulted in 280 mg of pgBTTT (71% yield).

2.2. Synthesis of homocoupling-free pgBTTT

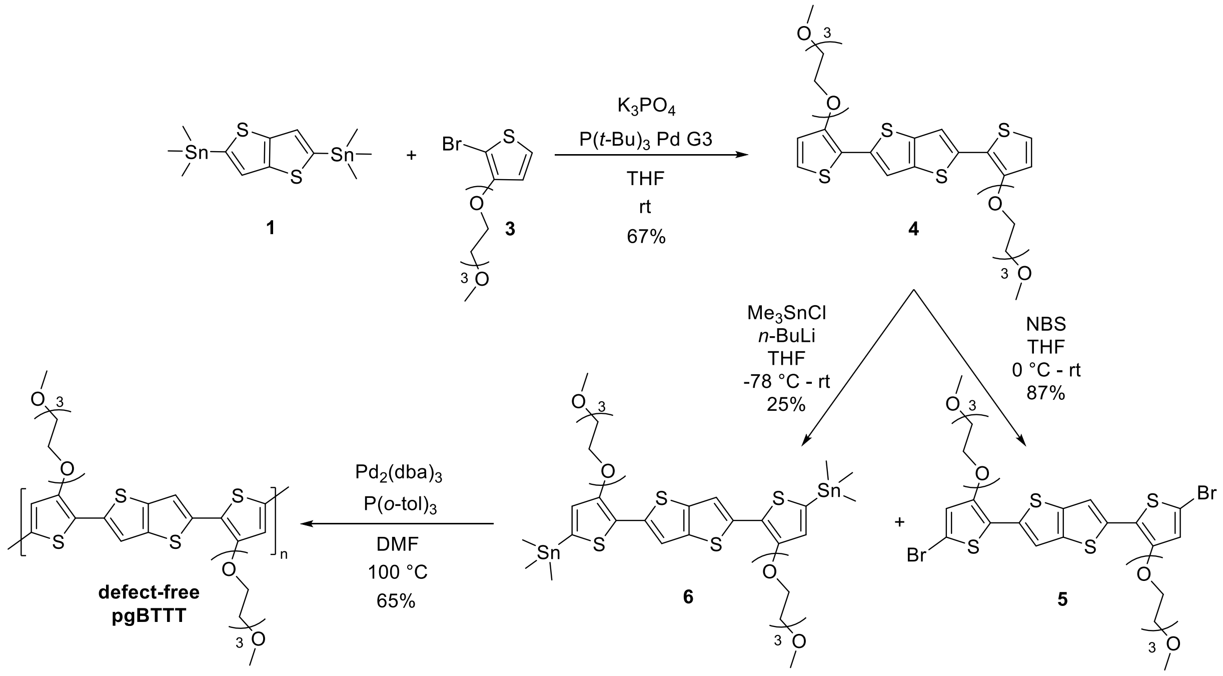

Scheme S2. Synthesis of homocoupling-free pgBTTT via the symmetrical Stille route.

2,5-bis(3-(2-(2-(2-methoxyethoxy)ethoxy)ethoxy)thiophen-2-yl)thieno[3,2-*b*]thiophene (**4**): Starting materials **1** and **3** were synthesized according to literature.[8,9] A schlenk tube was charged with compound **1** (0.77 g, 1.65 mmol, 1 eq.), glycolated thiophene **3** (1.29 g, 3.96 mmol, 2.4 eq.), potassium phosphate (351 mg, 1.65 mmol, 1 eq.), and $P(t\text{-}Bu)_3$ Pd G3 (47.3 mg, 82.6 µmol, 0.05 eq.), after which the tube was brought under inert atmosphere (using 5 vacuum/Ar cycles). Dry degassed THF (9 mL) was then added and the reaction was left to stir overnight at room temperature (rt). The crude reaction mixture was filtered over a silica plug with ethyl acetate and further purified via preparative GPC ($CHCl_3$) to obtain **4** as a brown oil (695 mg, 67%). ^{1}H NMR (400 MHz, $CDCl_3$): δ 7.38 (s, 2H), 7.06 (d, J = 5.5 Hz, 2H), 6.88 (d, J = 5.5 Hz, 2H), 4.32 – 4.26 (m, 4H), 3.93 – 3.89 (m, 4H), 3.78 – 3.75 (m, 4H), 3.70 – 3.64 (m, 8H), 3.55 – 3.51 (m, 4H), 3.36 (s, 6H).

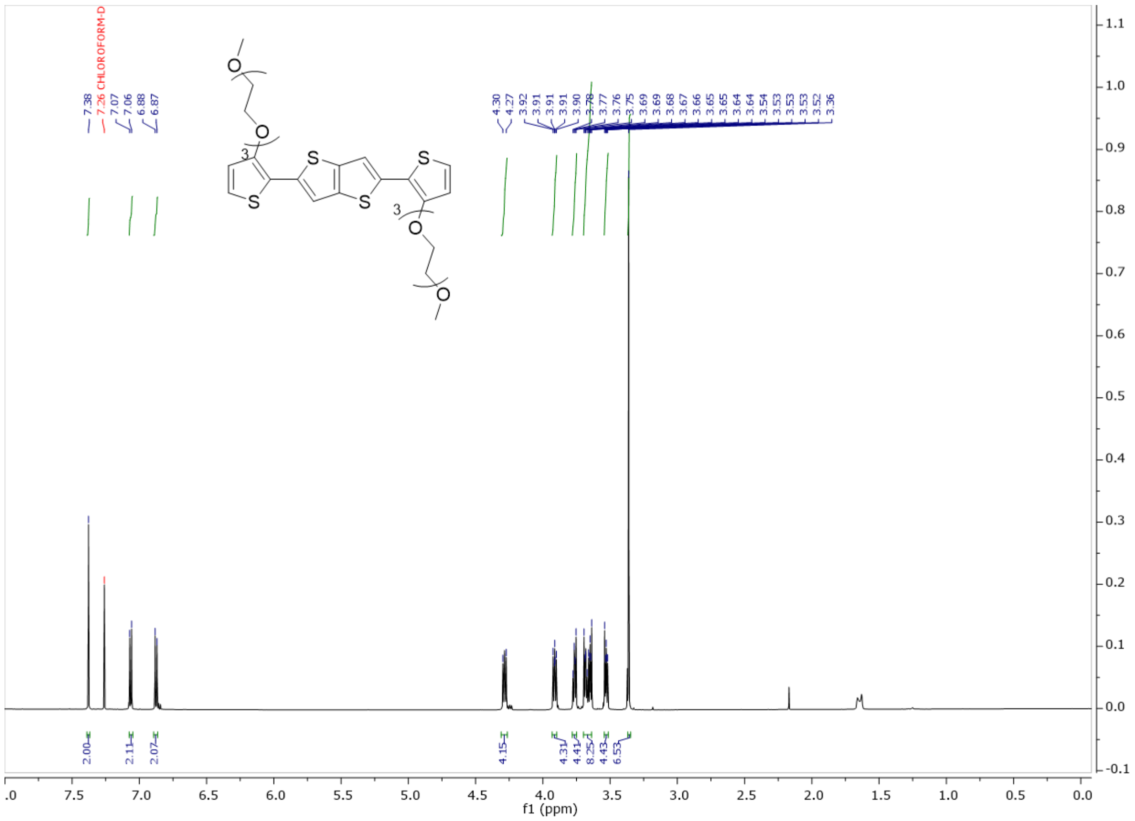

Figure S1. ^{1}H NMR spectrum of 2,5-bis(3-(2-(2-(2-methoxyethoxy)ethoxy)ethoxy)thiophen-2-yl)thieno[3,2-*b*]thiophene (**4**).

2,5-bis(5-bromo-3-(2-(2-(2-methoxyethoxy)ethoxy)ethoxy)thiophen-2-yl)thieno[3,2-*b*]thiophene (**5**): A round-bottom flask was charged with compound **4** (188 mg, 0.30 mmol, 1 eq.), which was dissolved in dry THF (8 mL) under inert argon atmosphere. The solution was cooled down to 0 °C and *N*-bromo-succinimide (106 mg, 0.60 mmol, 2 eq.) was added, after which the mixture was left to stir overnight at rt. The crude mixture was dissolved in diethyl ether, and washed 3 times with water and 1 time with brine. The organic phase was dried over $MgSO_4$ and filtered, after which the solvent was removed under reduced pressure. The product was purified *via* preparative GPC ($CHCl_3$) to obtain **5** as an orange oil (206 mg, 87% yield). ^{1}H NMR (400 MHz, $CDCl_3$): δ 7.26 (s, 2H), 6.90 (s, 2H), 4.26 – 4.22 (m, 4H), 3.90 – 3.86 (m, 4H), 3.76 – 3.72 (m, 4H), 3.69 – 3.63 (m, 8H), 3.55 – 3.52 (m, 4H), 3.37 (s, 6H). ^{13}C NMR (100 MHz, $CDCl_3$): δ 151.3, 138.5, 135.4, 121.7, 118.5, 115.2, 109.3, 72.1, 71.8, 71.1, 70.9, 70.8, 70.1, 59.2. MALDI-ToF MS [M^+]: m/z = 783.9.

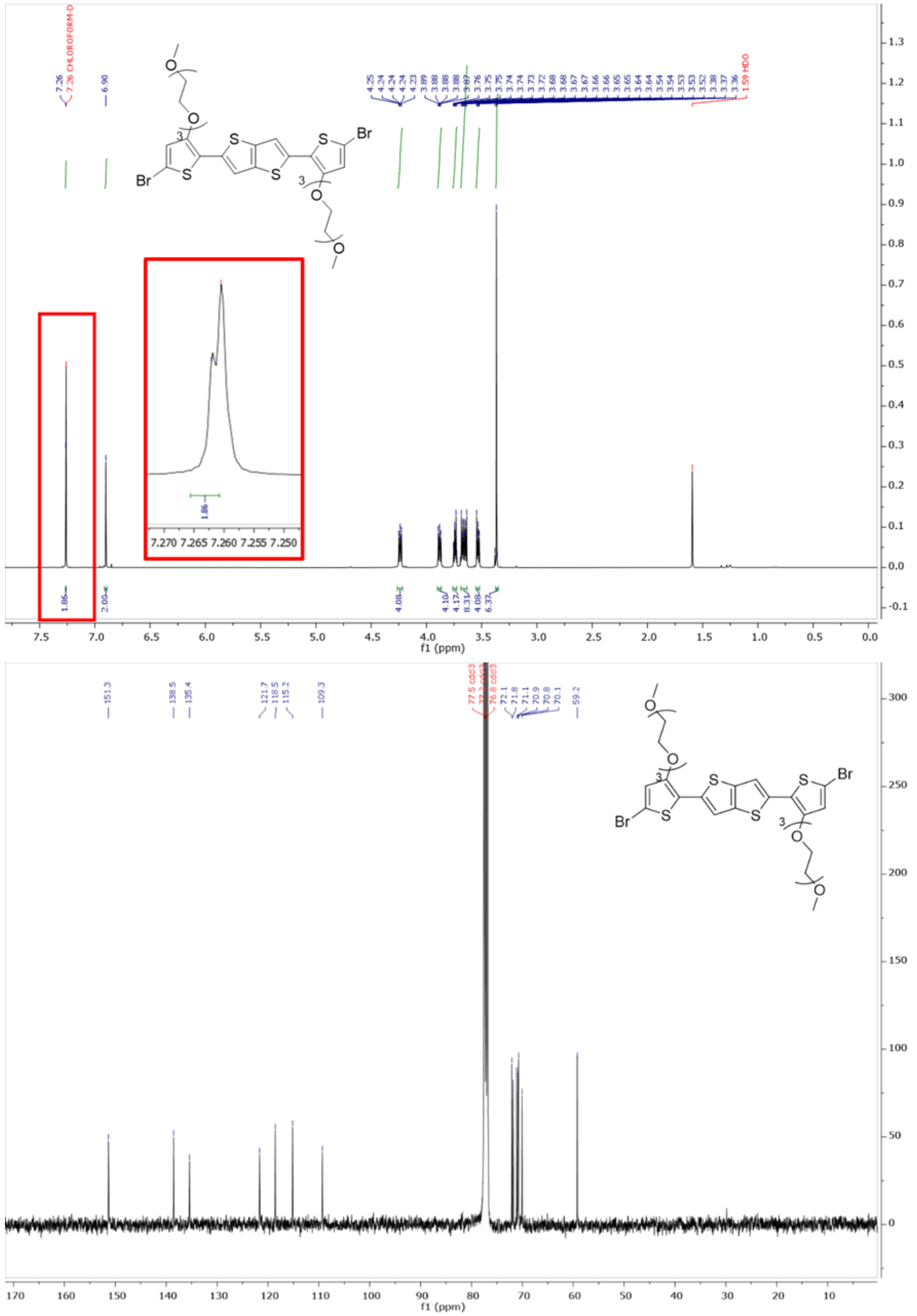

Figure S2. ^{1}H (top) and ^{13}C (bottom) NMR spectra of 2,5-bis(5-bromo-3-(2-(2-(2-methoxyethoxy)ethoxy)ethoxy)thiophen-2-yl)thieno[3,2-*b*]thiophene (**5**).

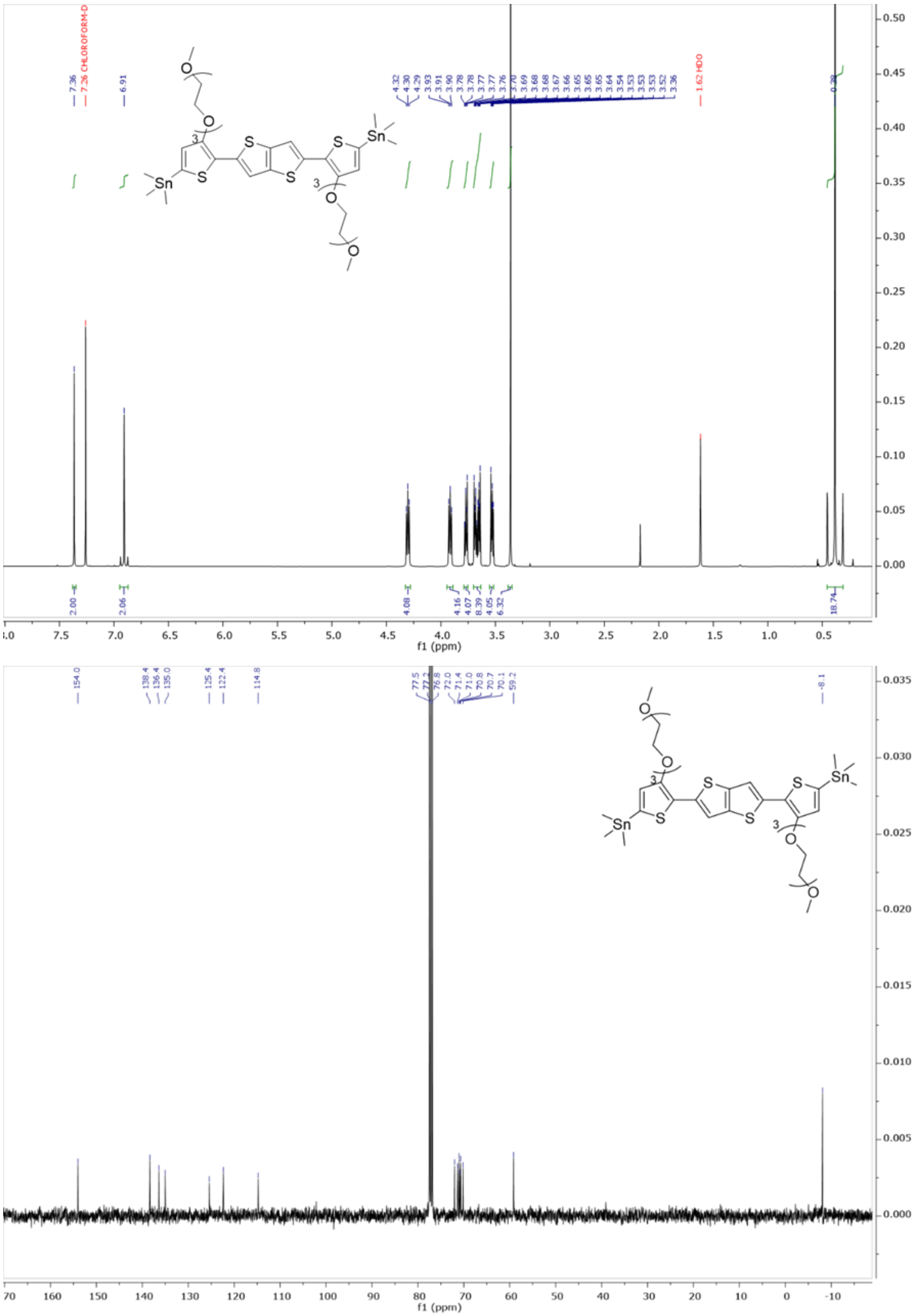

Figure S3. ^{1}H (top) and ^{13}C (bottom) NMR spectra of 2,5-bis(3-(2-(2-(2-methoxyethoxy)ethoxy)ethoxy)-5-(trimethyl-stannyl)thiophen-2-yl)thieno[3,2-*b*]thiophene (**6**).

2,5-bis(3-(2-(2-(2-methoxyethoxy)ethoxy)ethoxy)-5-(trimethylstannyl)thiophen-2-yl)thieno-[3,2-*b*]-thiophene (**6**): A round-bottom flask was charged with compound **4** (500 mg, 0.795 mmol, 1 eq.), which was dissolved in dry THF (15 mL) under inert argon atmosphere. The solution was cooled down to −78 °C with liquid nitrogen and *n*-butyllithium (2.5 M in hexanes; 765 µL, 1.91 mmol, 2.4 eq.) was added dropwise. The mixture was allowed to warm up gently to –20 °C, after which it was cooled again to −78 °C before adding trimethyltin chloride (1 M; 238 µL, 2.38 mmol, 3 eq.). The solution was left to stir overnight while slowly warming up to rt. Afterwards, water was added and the crude mixture was extracted with $CHCl_3$, dried over $MgSO_4$, and filtered. The solvent was removed *via* rotary evaporation and the product was purified *via* preparative GPC ($CHCl_3$) to obtain distannylated monomer **6** as a brown oil (189 mg, 25% yield). ^{1}H NMR (400 MHz, $CDCl_3$): δ 7.36 (s, 2H), 6.91 (s, 2H), 4.33 – 4.28 (m, 4H), 3.94 – 3.89 (m, 4H), 3.79 – 3.75 (m, 4H), 3.70 – 3.63 (m, 8H), 3.55 – 3.52 (m, 4H), 3.36 (s, 6H), 0.38 (s, 18H). ^{13}C NMR (100 MHz, $CDCl_3$): δ 154.0, 138.4, 136.4, 135.0, 125.4, 122.4, 114.8, 72.0, 71.4, 71.0, 70.8, 70.7, 70.1, 59.2, −8.1.

Homocoupling-free pgBTTT: A schlenk tube was charged with monomer **5** (100 mg, 127 µmol, 1 eq.), monomer **6** (121 mg, 127 µmol, 1 eq.), tris(dibenzylideneacetone)dipalladium(0) (2.3 mg, 2.5 µmol, 0.02 eq.), tri(*o*-tolyl)phosphine (3.1 mg, 10.2 µmol, 0.08 eq.), and 3 mL of anhydrous degassed DMF. The schlenk tube was then brought under inert atmosphere (using 5 vacuum/Ar cycles) and heated overnight at 100 °C. Afterwards, the mixture was diluted with DMF, the Pd scavenger diethylammonium diethyldithiocarbamate was added, and the mixture was stirred for 1 h at 100 °C. Subsequently, the mixture was added to 50 mL of cold methanol. The crude polymer precipitate was filtered off and subjected to Soxhlet extractions with methanol, acetone, hexanes, THF, dichloromethane (DCM), and chloroform. The DCM fraction was dried under Ar flow and afterwards high vacuum, which resulted in 21 mg of polymer (13% yield). The chloroform fraction was evaporated to a minimum volume and once again added to methanol. The precipitate was filtered off and dried under high vacuum. This resulted in 104 mg of homocoupling-free pgBTTT (65% yield).

The synthesis was repeated twice, producing chloroform fractions in 63 and 64% yield, respectively.

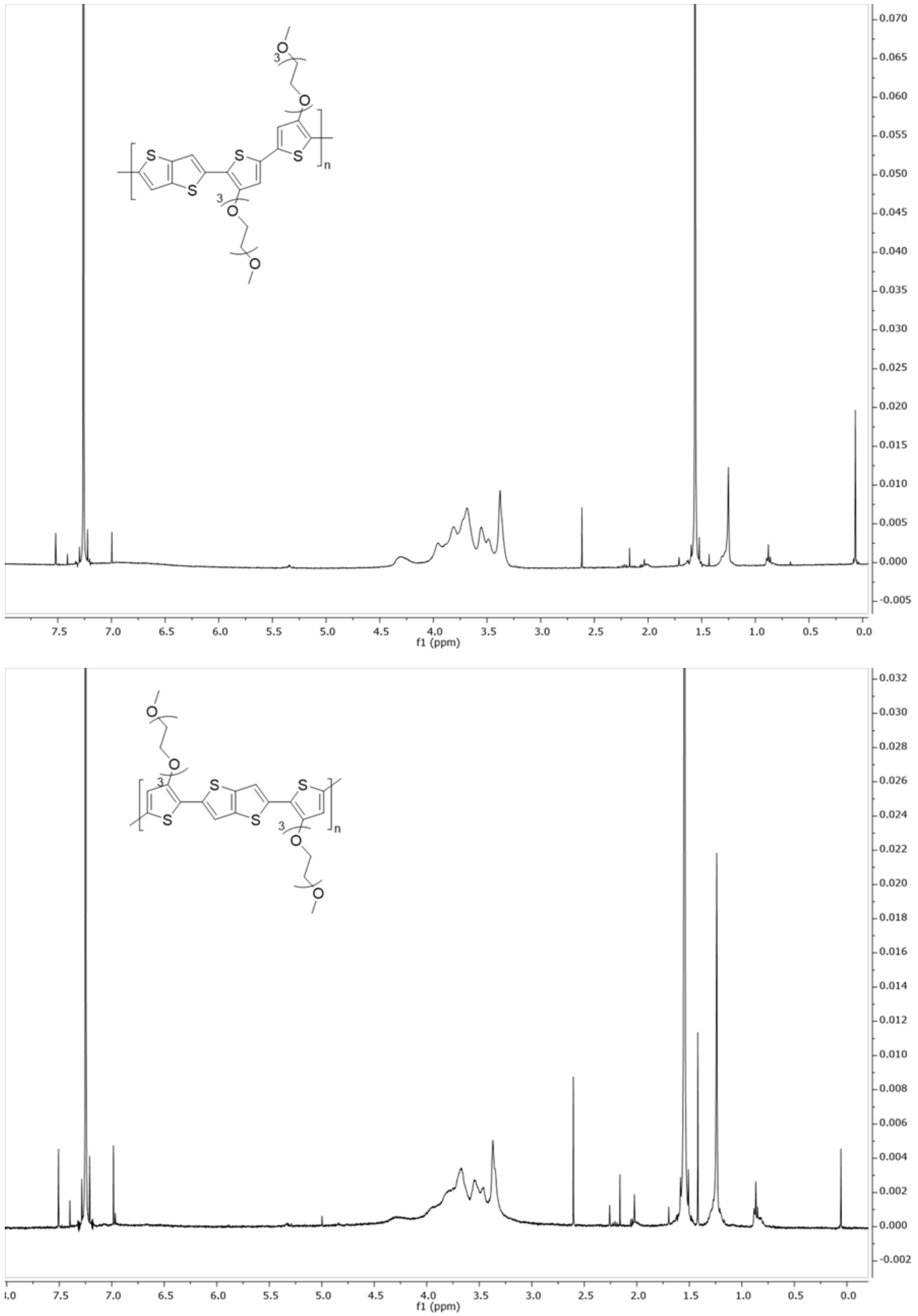

Figure S4. ^{1}H NMR spectra of regular pgBTTT (chloroform fraction, top) and homocoupling-free pgBTTT (chloroform fraction, bottom).

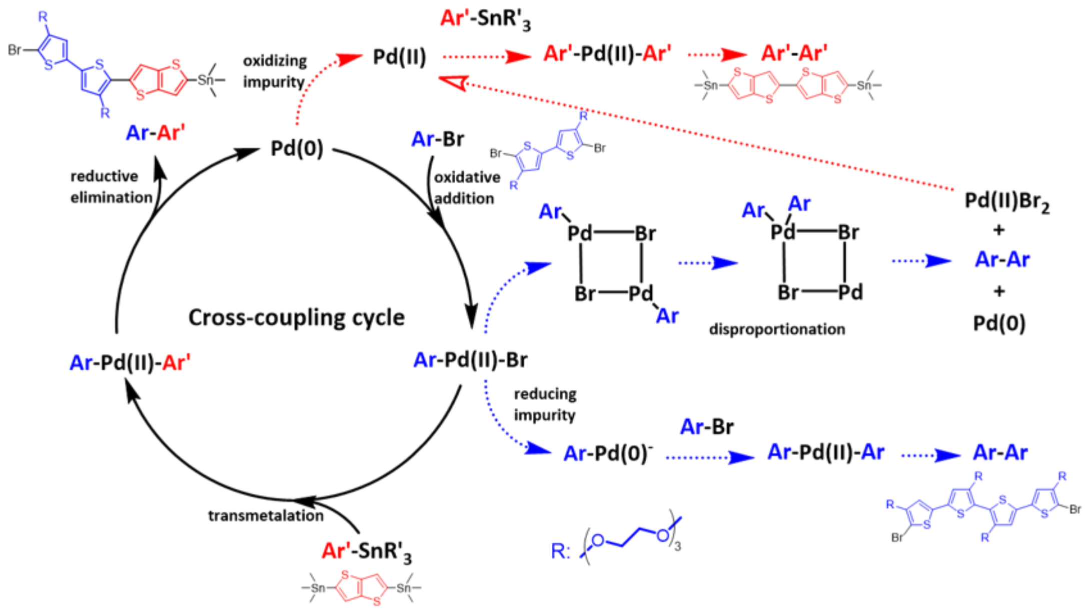

Scheme S3. Simplified overview of the Stille catalytic cycle and the side reactions leading to homocoupling sequences in the conventional pgBTTT synthesis (adapted from ref. [3]).

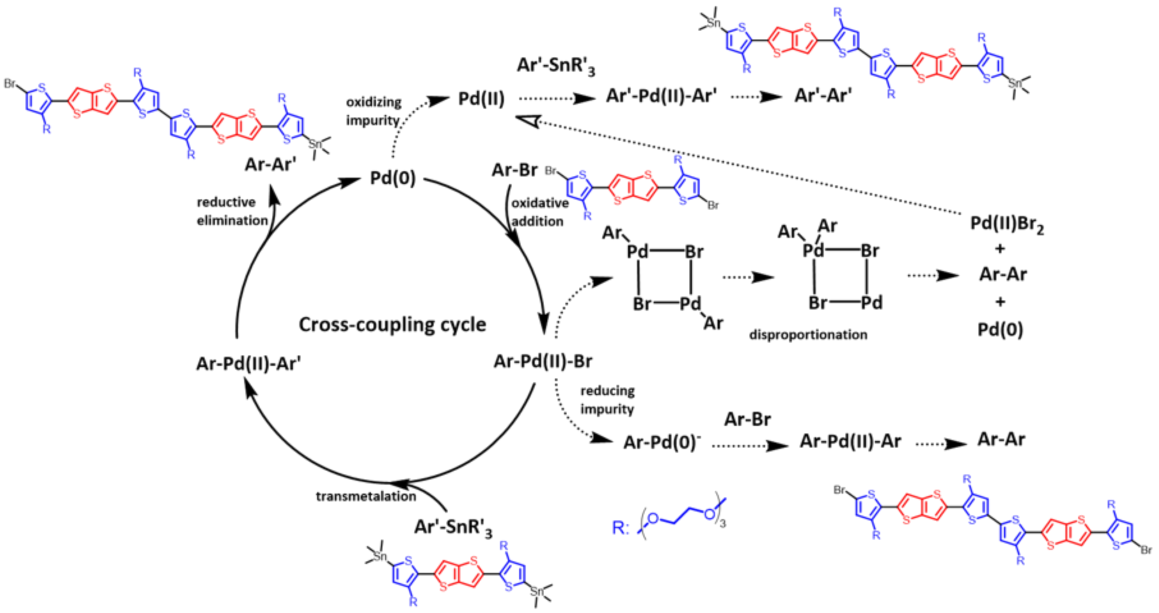

Scheme S4. Simplified overview of the Stille catalytic cycle and the homocoupling side reactions (adapted from ref. [3]) which, when using a symmetrical monomer, result in the exact same (homocoupling-free) pgBTTT structure.

2.3. Optimization efforts toward homocoupling-free pgBTTT

The synthesis of homocoupling-free pgBTTT required some optimization. Initially, the P(*t*-Bu)$_3$ Pd G3 catalytic system was tested in a rt version of the standard Stille polymerization (**Figure S5.A**).[11] However, MALDI-ToF MS analysis revealed persisting homocoupling defects, with off-ratios between the bithiophene and thienothiophene monomers that were even larger than for the regularly synthesized polymer (**Figure S9-S10**), rendering this method unsuitable for homocoupling-free pgBTTT synthesis.

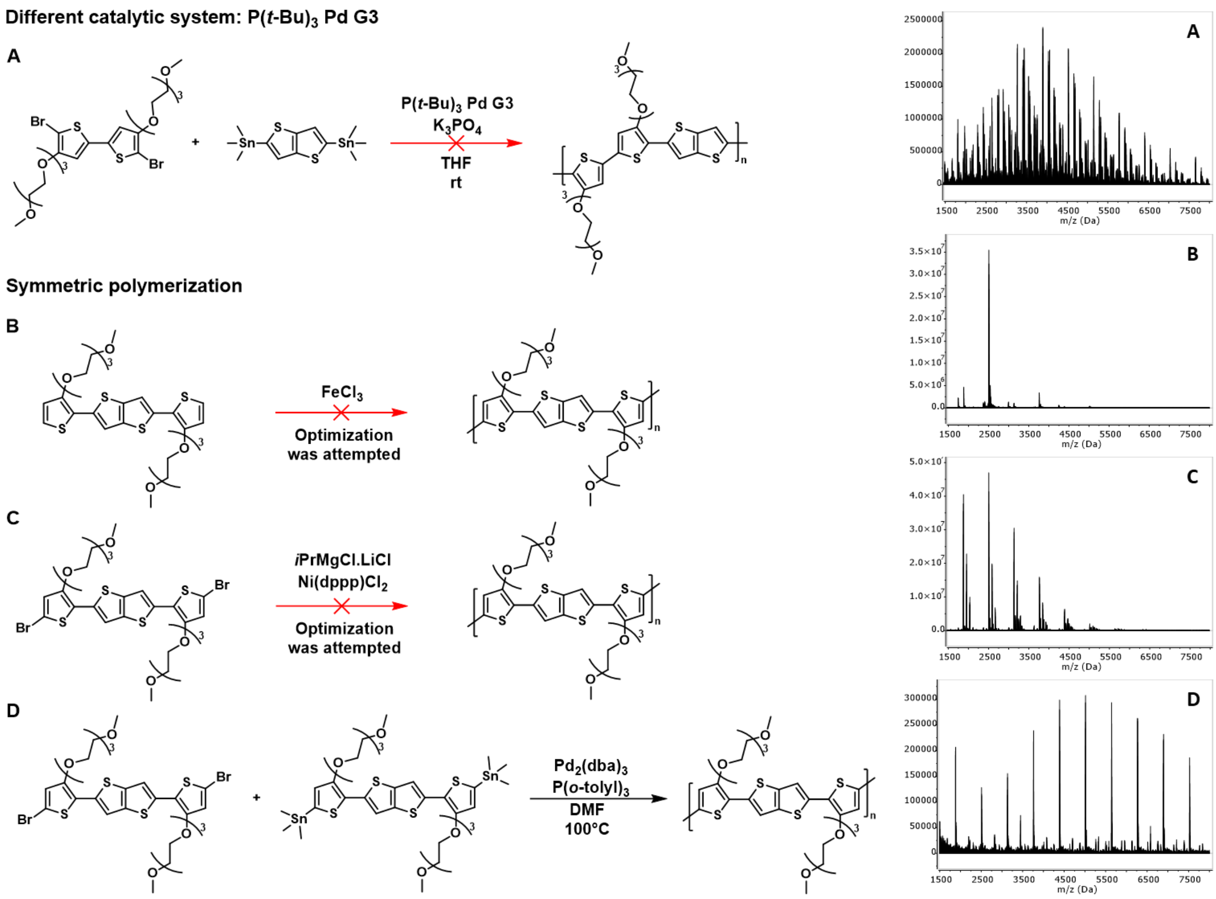

Figure S5. Different routes explored for the synthesis of homocoupling-free pgBTTT (left) and the accompanying MALDI-ToF mass spectra (*m/z* = 1.5 to 8 kDa) for the highest molar mass fractions (right). When optimization was attempted, the mass spectrum after optimization is shown.

A second attempt already exploited the symmetric nature of pgBTTT, as previously done for pBTTT-(OR)$_2$.[12] Hence, instead of using 2 monomers, 1 symmetric monomer was designed, containing a thienothiophene core flanked by glycolated thiophenes. At first, this monomer was oxidatively polymerized using iron(III) chloride ($FeCl_3$), resulting in a polymer with

exclusively 1:1 ratios of gBT:TT, as confirmed by MALDI-ToF MS (**Figure S5.B** and **S11**). Unfortunately, this relatively straightforward polymerization method afforded insoluble/non-processable polymers. The poor solubility in common organic solvents led to substantial losses during Soxhlet extraction. Consequently, the polymer fractions that could be recovered from the Soxhlet extraction showed only a few couplings, based on MALDI-ToF MS, while the rest of the material remained in the thimble.

Initially, we hypothesized that the poor processability originated from excessively high molar masses or partial branching/crosslinking. Therefore, we first varied the polymerization solvent and temperature in an attempt to obtain lower-molar-mass, more soluble fractions (**Table S1**, entries 1–5). These attempts consistently yielded largely insoluble material. Subsequent UV-Vis-NIR and cyclic voltammetry measurements revealed that the oxidative polymerization led to extensive doping of the polymer, evident from the pronounced polaron signal in the UV-Vis-NIR absorption spectrum and a significant shift of the oxidation onset (to lower values) in the aqueous cyclic voltammogram (**Figure S6**).[13,14] Also, an unusually high iron content (18000 ppm) was detected *via* inductively coupled plasma mass spectrometry (ICP-MS). This prompted us to focus on post-polymerization dedoping and metal scavenging strategies (**Table S1**, entries 6–11).

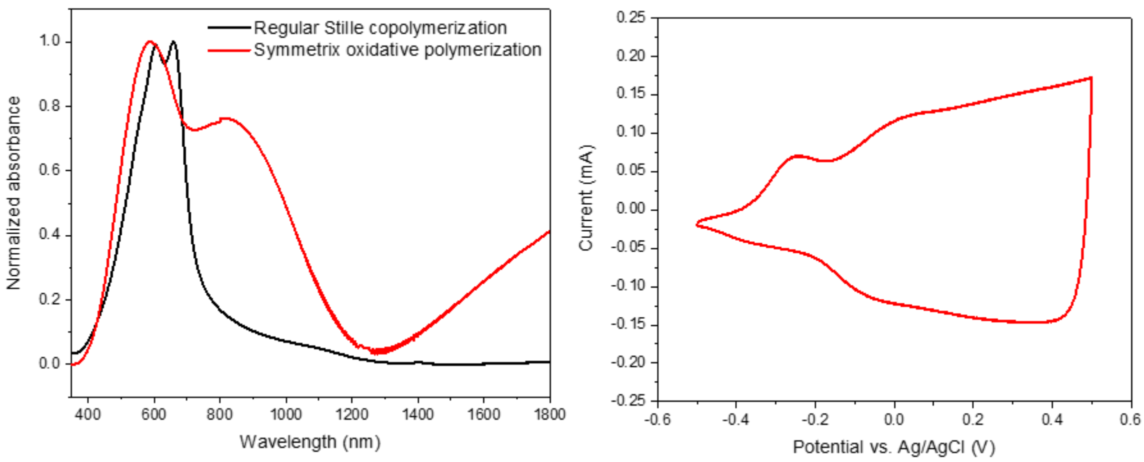

Figure S6. Normalized UV-Vis-NIR absorption spectra (left) of regular pgBTTT (black) and homocoupling-free pgBTTT synthesized *via* oxidative polymerization (red). Aqueous cyclic voltammetry spectra (scans 2-5) of homocoupling-free pgBTTT synthesized *via* oxidative polymerization (right), measured at a scan rate of 100 mV s^{-1} in aqueous electrolyte (0.1 M NaCl in distilled water). The oxidation onsets of the first and second oxidation peaks are situated at –0.40 and –0.30 V, respectively.

Table S1. Optimization attempts for the oxidative polymerization ($FeCl_3$) and subsequent scavenging/dedoping of pgBTTT.

	Solvent[a)]	Temperature	Scavenging/dedoping		Outcome	
			System[b)]	Stage	Processability	Doping state
1	ODCB	50 °C	/	/	Dominant insoluble fraction	n.d.[c)]
2	CB	50 °C	/	/	Dominant insoluble fraction	n.d.
3	DMF	50 °C	/	/	Reaction failed	n.d.
4	CB	22 °C	/	/	Dominant insoluble fraction	n.d.
5	$CHCl_3$	22 °C	/	/	Dominant insoluble fraction	n.d.
6	ODCB	100 °C	EDTA + Diethylammonium diethyldithiocarbamate	Add to crude[d)] Stir, 1 h, 100 °C	Dominant insoluble fraction	Last Soxhlet fraction is doped[e)]
7	ODCB	100 °C	Aq. solution of EDTA and Et_3N	After Soxhlet extractions Stir, 4 h, 60 °C	Dominant insoluble fraction	Last Soxhlet fraction is doped[e)]
8	CB	50 °C	Aq. solution of EDTA and Et_3N	Add to crude polymer mixture Stir, 4 h, 60 °C	Dominant insoluble fraction	Last Soxhlet fraction is doped[e)]
9	CB	50 °C	Aq. solution of EDTA and Et_3N	Add to crude polymer mixture Stir, overnight, 60 °C	Dominant insoluble fraction	Last Soxhlet fraction is doped[e)]
10	CB	50 °C	Aq. solution of EDTA and Et_3N	First precipitate crude polymer and filter Stir, overnight, 60 °C	Dominant insoluble fraction	Last Soxhlet fraction is dedoped[e)]
11	CB	50 °C	Aq. solution of EDTA	First precipitate crude polymer and filter Stir, overnight, 60 °C	Dominant insoluble fraction	Last Soxhlet fraction is dedoped[e)]

[a)] ODCB = *ortho*-dichlorobenzene, CB = chlorobenzene, DMF = dimethylformamide, $CHCl_3$ = chloroform. [b)] EDTA = ethylenediaminetetraacetic acid, Et_3N = triethylamine. [c)] n.d. = not determined. [d)] Standard scavenging procedure for conjugated polymers. [e)] As evidenced by the presence/absence of polaronic bands in the UV-Vis-NIR absorption spectra.

A range of scavenging and dedoping protocols using EDTA (to remove Fe species) and triethylamine (to chemically dedope the polymer) were applied at different stages of the work-up (**Table S1**). While some of these treatments successfully reduced or eliminated the polaronic absorption features in UV-Vis-NIR spectra (entries 10–11), the resulting materials remained poorly soluble and could not be processed further. This indicates that dedoping alone is not

sufficient to restore solubility. As a result, the oxidative polymerization route was abandoned and alternative strategies were explored.

Another common route toward thiophene-containing conjugated polymers is the Grignard metathesis (GRIM) polymerization.[15,16] In this method, a dibrominated monomer is treated with one equivalent of an alkyl Grignard reagent, which results in a magnesium-bromine exchange via a GRIM reaction. The resulting monomer is then polymerized using a nickel catalyst. To this end, the previously used symmetric monomer underwent bromination prior to GRIM polymerization (**Figure S5.C**). Initial MALDI-ToF MS analysis of the GRIM-pgBTTT showed rather short chains consisting of a 1:1 gBT-TT ratio with some reactive bromine end-groups, suggesting the reaction could potentially be pushed to higher molar masses (**Figure S12**). However, despite optimization efforts (involving reaction solvents, temperature, and duration), obtaining sufficiently high molar mass fractions proved elusive.

In a final attempt to achieve defect-free pgBTTT, the symmetric polymerization was executed *via* a Stille route, with one portion of the symmetric monomer being brominated and the other one stanylated (**Figure S5.D**). Subsequent Stille polymerization can therefore only result in the target structure. Through this method, a high molar mass batch of homocoupling-free pgBTTT was ultimately obtained.

In **Figure S13** and **S14**, the MALDI-ToF mass spectra of different molar mass fractions of homocoupling-free pgBTTT are shown. These fractions were obtained *via* Soxhlet extraction, a technique used for polymer purification by selectively dissolving and removing low-molar-mass species from a bulk polymer sample. In this process, a solvent is continuously evaporated, condensed, and percolated through the polymer in a Soxhlet thimble, dissolving the more soluble fractions while leaving behind the higher molar mass chains. This repeated solvent cycling ensures efficient separation and molar mass fractionation. After the symmetric synthesis of pgBTTT, consecutive Soxhlet extractions were performed with methanol, acetone, hexanes, THF, DCM, and chloroform. So, while the highest molar mass fraction of homocoupling-free pgBTTT eluted in chloroform, the DCM fraction contains the second highest molar mass chains.

3. MALDI-ToF MS

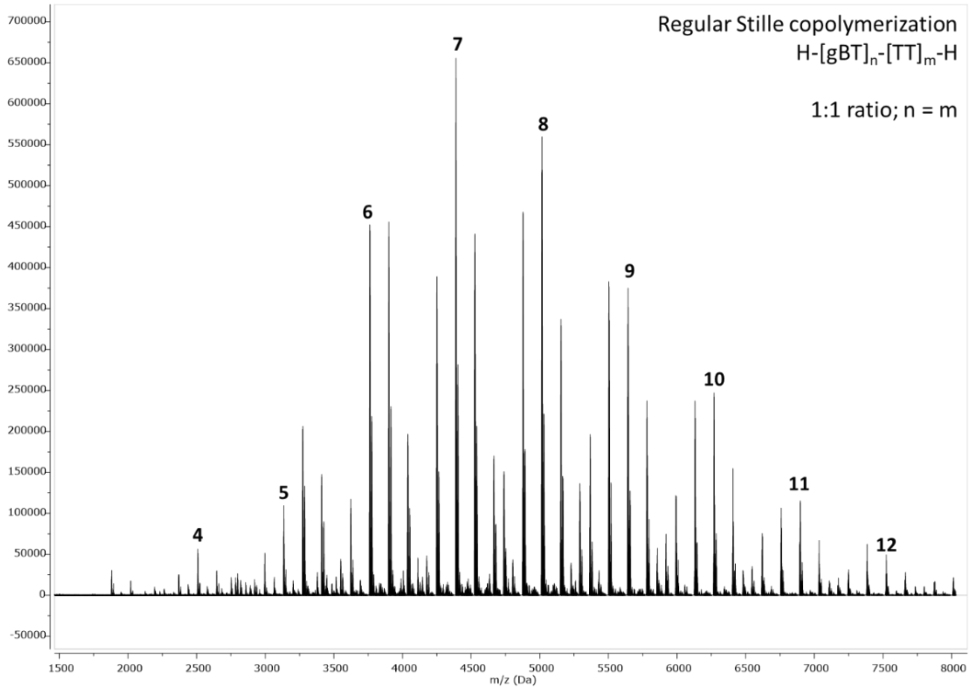

Figure S7. MALDI-ToF mass spectrum of conventional pgBTTT synthesized *via* the standard Stille copolymerization. The signals representing species containing a 1:1 gBT:TT ratio are annotated with the number of gBTTT units above the respective peaks.

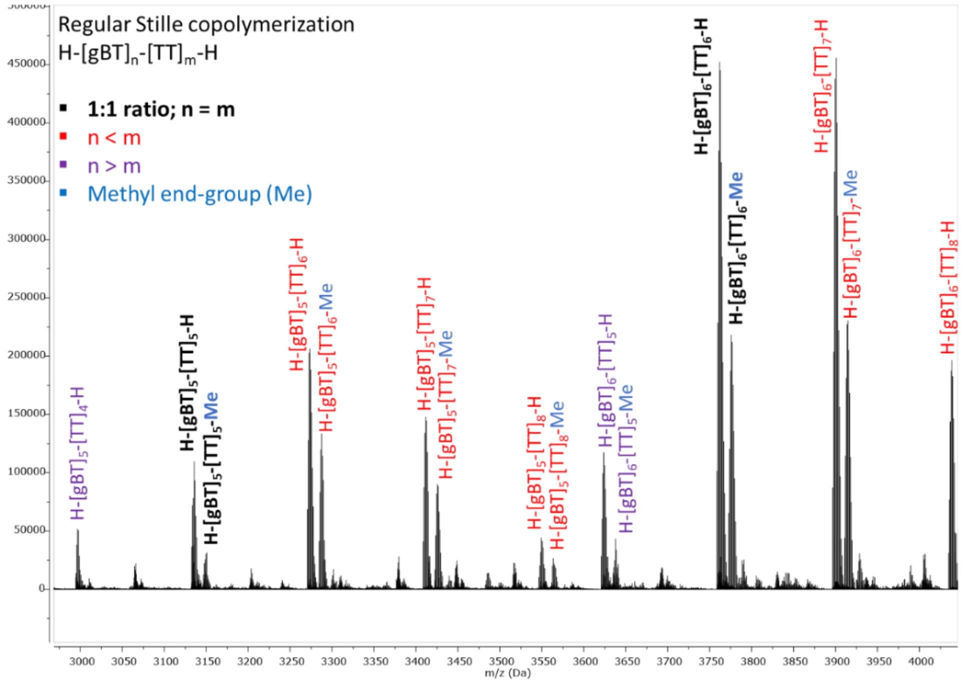

Figure S8. Excerpt of the MALDI-ToF mass spectrum of conventional pgBTTT synthesized *via* the standard Stille copolymerization in the 3–4 kDa range with the annotations showing 1:1 gBT:TT ratios (black), deviating gBT:TT ratios (red/purple), and different end-groups (blue).

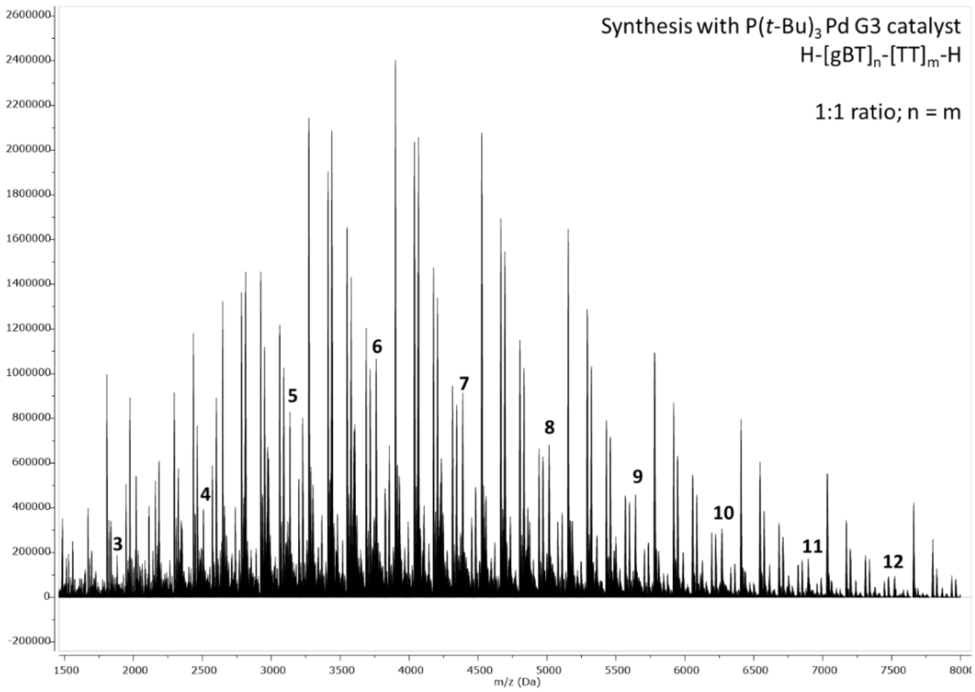

Figure S9. MALDI-ToF mass spectrum of pgBTTT synthesized *via* room temperature Stille cross-coupling with the P(*t*-Bu)3 Pd G3 catalyst. The signals representing species containing a 1:1 gBT:TT ratio are annotated with the number of gBTTT units above the respective peaks.

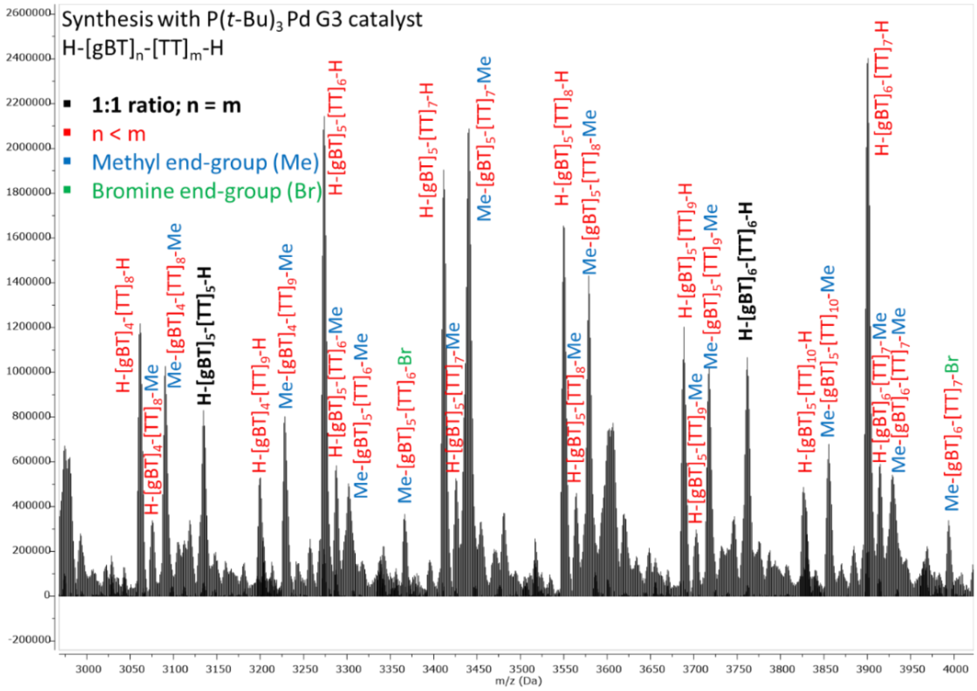

Figure S10. Excerpt of the MALDI-ToF mass spectrum of pgBTTT synthesized *via* room temperature Stille cross-coupling with the P(*t*-Bu)3 Pd G3 catalyst in the 3–4 kDa range with the annotations showing 1:1 gBT:TT ratios (black), deviating gBT:TT ratios (red), and different end-groups (blue/green).

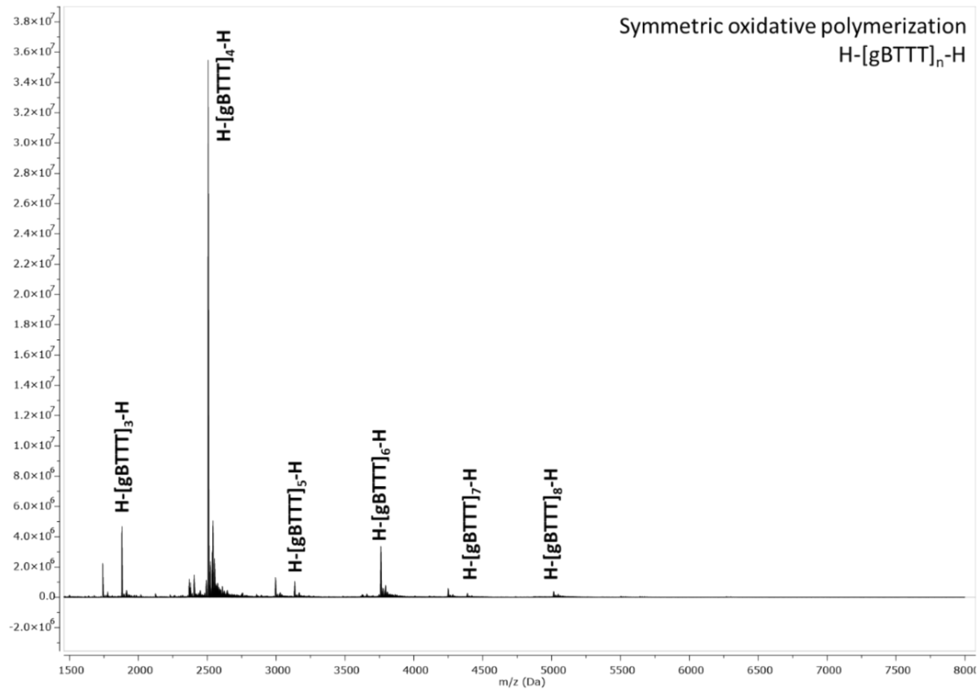

Figure S11. MALDI-ToF mass spectrum of pgBTTT synthesized *via* oxidative polymerization using $FeCl_3$. The signals representing species containing a 1:1 gBT:TT ratio are annotated using H-[gBTTT]$_n$-H, with n representing the number of gBTTT units, above the respective peaks.

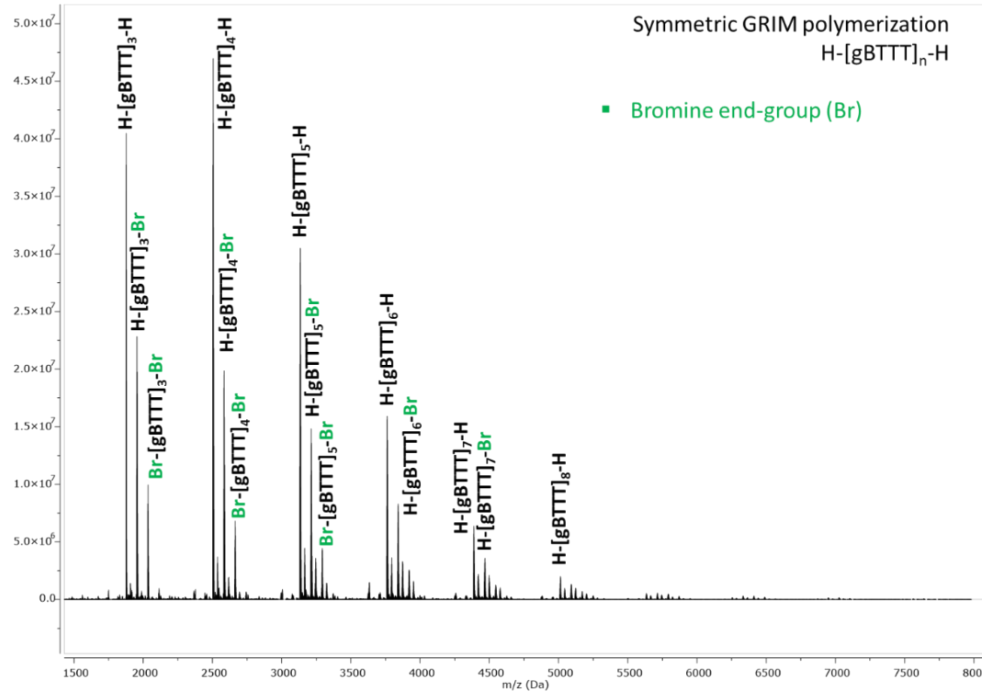

Figure S12. MALDI-ToF mass spectrum of pgBTTT synthesized *via* GRIM polymerization. The signals representing species containing a 1:1 gBT:TT ratio are annotated using H-[gBTTT]$_n$-H, with n representing the number of gBTTT units, above the respective peaks. Bromine end-groups are indicated in green.

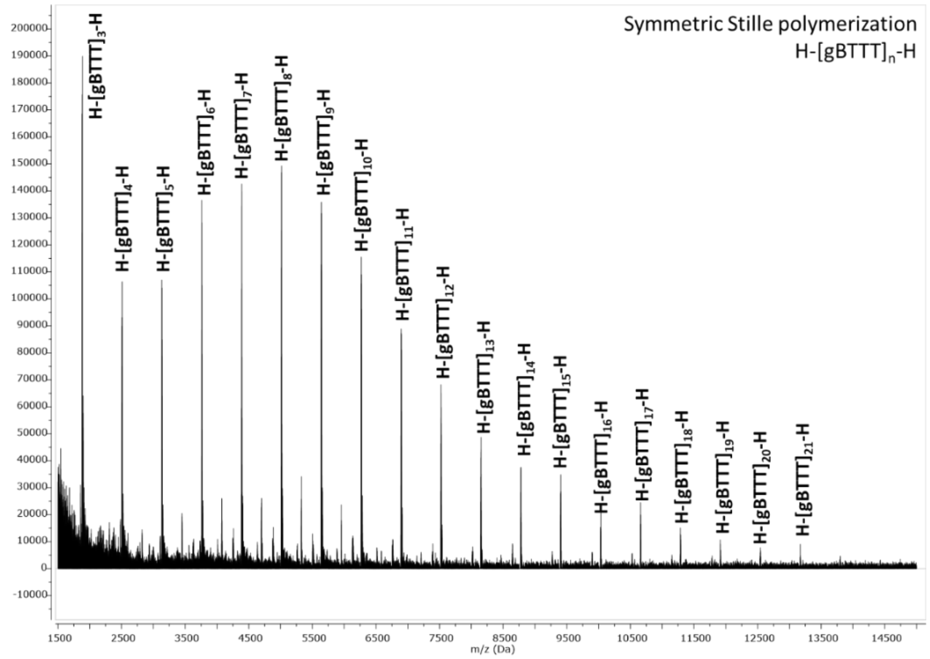

Figure S13. MALDI-ToF mass spectrum of homocoupling-free pgBTTT (chloroform fraction) synthesized *via* symmetric Stille polymerization. The signals representing species containing a 1:1 gBT:TT ratio are annotated using H-[gBTTT]$_n$-H, with n representing the number of gBTTT units, above the respective peaks. A second distribution with lower intensity is visible as well, which corresponds to species that are charged twice (z = 2).

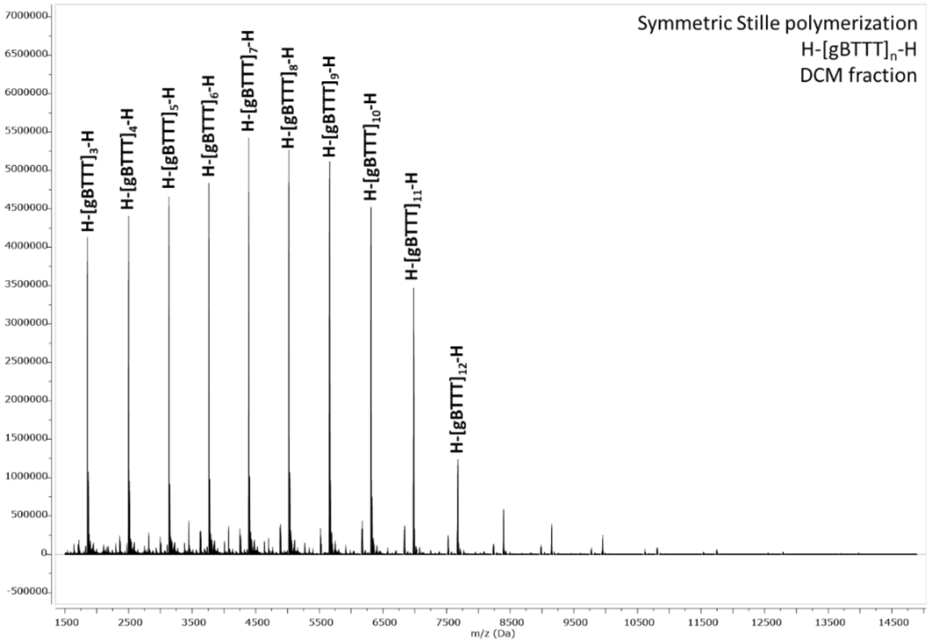

Figure S14. MALDI-ToF mass spectrum of homocoupling-free pgBTTT (lower molar mass DCM fraction) synthesized *via* symmetric Stille polymerization. The signals representing species containing a 1:1 gBT:TT ratio are annotated using H-[gBTTT]$_n$-H, with n representing the number of gBTTT units, above the respective peaks.

4. UV-Vis-NIR absorption spectroscopy

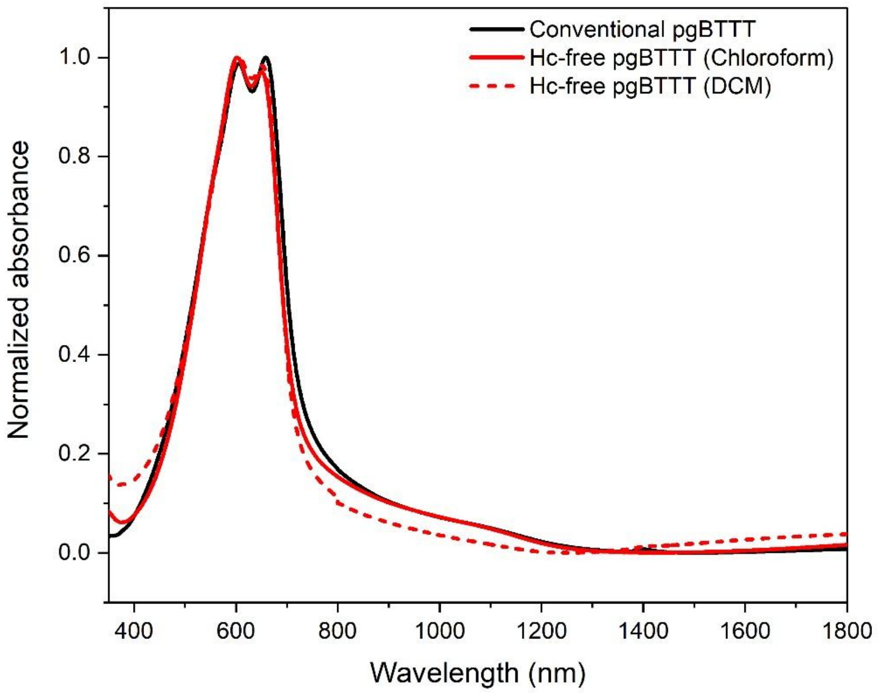

Figure S15. Normalized UV-Vis-NIR absorption spectra of conventional (black) and homocoupling-free (hc-free) pgBTTT (red) as obtained from the chloroform (solid line) and DCM (dashed line) Soxhlet fractions.

5. Cyclic voltammetry

Table S2. Summary of the electrochemical properties of conventional and homocoupling-free (hc-free) pgBTTT.

	$HOMO_{org}$[a] (eV)	$LUMO_{org}$[a] (eV)	$E_{ox,org}$[a] (V)	$E_{ox,aq}$[b] (V)
Conventional pgBTTT	−4.49	−2.88	−0.42	−0.09
Hc-free pgBTTT $CHCl_3$ fraction	−4.50	−2.89	−0.40	−0.08
Hc-free pgBTTT DCM fraction	−4.50	−2.86	−0.40	−0.08

[a] Electrochemically determined using a solution of 0.1 M tetrabutylammonium hexafluorophosphate in acetonitrile and ferrocene as the external standard. [b] Determined using a 0.1 M aqueous sodium chloride solution.

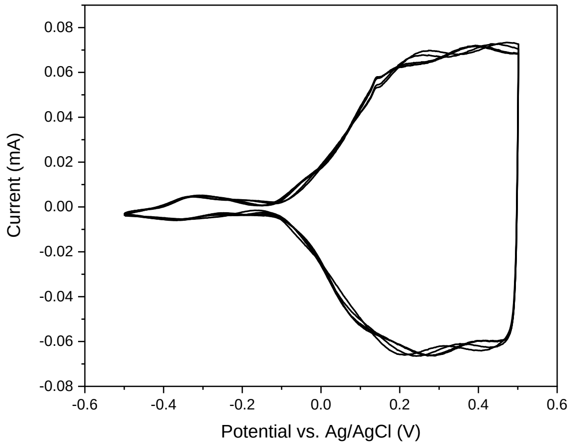

Figure S16. Cyclic voltammograms (scans 2–6) for conventional pgBTTT, measured at a scan rate of 100 mV s^{-1} in aqueous electrolyte (0.1 M NaCl in distilled water).

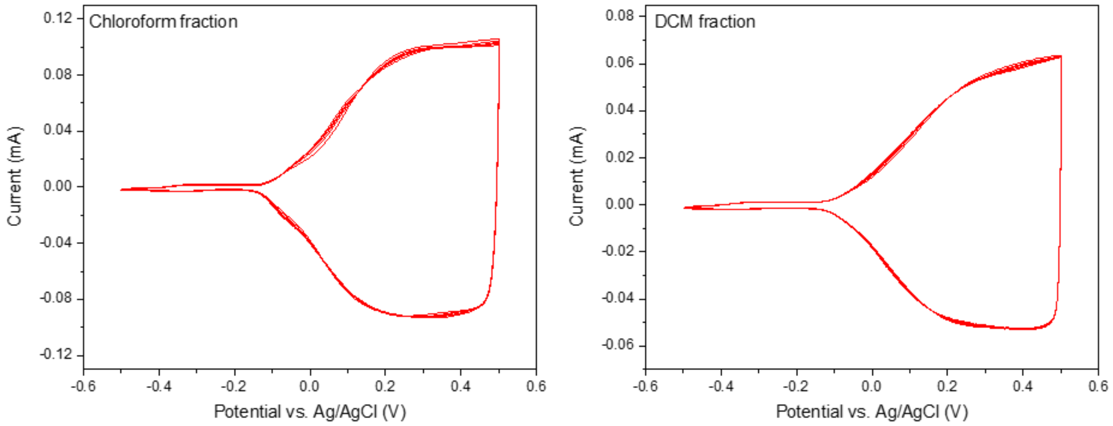

Figure S17. Cyclic voltammograms (scans 2–6) for homocoupling-free pgBTTT – chloroform fraction (left) and DCM fraction (right) – measured at a scan rate of 100 mV s^{-1} in aqueous electrolyte (0.1 M NaCl in distilled water).

6. ESD-STM

6.1. Analysis of defects and their correlations in polymer sequences

The presence and frequency of polymerization defects were investigated for both homocoupling-free and conventional pgBTTT. The analysis was performed on a selection of about 10 highly resolved STM images per batch, fitted with energy-minimized molecular models of the repeat units, to determine the exact sequence of the backbones. Possible relative *trans* or *cis* conformations of the glycolated thiophene (gT) and thienothiophene (TT) subunits were taken into account. While we observed homocoupling defects of both comonomers in conventional pgBTTT, no defects were identified in the symmetric Stille polymer. The bond statistics for the conventional pgBTTT sample are reported in **Figure 2**, while the statistics for the symmetric Stille polymer were derived from analyzing approximately 450 bonds between (gT-TT-gT) monomers.

Access to the precise sequence of defective and defect-free polymers enables to investigate the role of defects in the polymerization process. Literature suggests that the formation of homocoupling defects influences − typically impedes or hinders − the growth of polymers.[17] In order to determine if the presence of defects affects the growth of the polymeric chain in conventional pgBTTT, we examined whether the defects are uniformly distributed throughout the polymer chains and whether polymer length influences defect density. In particular, we performed the three following statistical tests:

i) a correlation analysis to examine the relationship between consecutive defects;
ii) an evaluation of defect density as a function of polymer length;
iii) an assessment of defect density at the polymer ends.

6.1.1. Analysis of the correlation between consecutive defects

The first step was to determine whether homocoupling defects were randomly distributed within the polymers or if they exhibited a tendency to cluster, which could suggest that the presence of a coupling defect influences the polymerization process and increases the likelihood of additional defects.

To this end, we analyzed consecutive pairs of bonds between TT and glycolated bithiophene (gBT) monomers, examining a large number of blocks of three consecutive monomers. Each TT-gBT bond was labelled as "o", representing a regular coupling, while each homocoupling (TT-TT or gBT-gBT) was labeled as "h". For simplicity, we classified all homocoupling defects under a single category, as TT-TT and gBT-gBT homocouplings were observed to occur at similar frequencies. We recorded the number of occurrences for three specific sequences:

consecutive defects ("hh"), consecutive regular alternating bonds ("oo"), and mixed sequences where a defect was adjacent to a regular bond ("ho/oh"). The results of this analysis are presented in **Table S3** (left, observed values).

Next, we tested the null hypothesis that the presence of an "o" or "h" bond in the chain is independent of the presence (or absence) of a defect in the neighboring bond, implying that consecutive bonds are uncorrelated. This hypothesis was assessed by calculating the probabilities for the three possible pairwise sequences (*i.e.* "hh," "ho/oh," and "oo") under the assumption of independence, and comparing these probabilities with the experimentally observed frequencies.

Under the assumption that consecutive bonds are independent, the probabilities for the sequences "hh", "ho/oh", or "oo" can be calculated as follows:

$$\mathrm{Pr(hh)} = \mathrm{Pr(h)}\,\mathrm{Pr(h)} = \mathrm{Pr\,(h)}^2 \quad (1)$$

$$\mathrm{Pr(ho/oh)} = 2\,\mathrm{Pr(h)}\,\mathrm{Pr(o)} = 2\,\mathrm{Pr(h)}\,[1 - \mathrm{Pr\,(h)}] \quad (2)$$

$$\mathrm{Pr(oo)} = \mathrm{Pr(o)}\,\mathrm{Pr(o)} = [1 - \mathrm{Pr\,(h)}]^2 \quad (3)$$

where we have used the relation $\mathrm{Pr(o)} = 1 - \mathrm{Pr(h)}$ and the assumptions of independence.

The probabilities (1)-(3) are functions of a single parameter (or degree of freedom), $\mathrm{Pr(h)}$, which represents the probability of finding a homocoupling defect. Given the large number of examined bonds, this probability can be estimated as the measured relative frequency of homocoupling defects (of either type). Based on the statistics reported in **Figure 2**, we can thus estimate that $\mathrm{Pr(h)} = 0.09$.

The resulting probabilities (1)-(3) and the corresponding expected counts are presented in **Table S3** (right, expected values). These values were compared with the experimentally obtained counts in **Table S3** (left, observed values) and the validity of the null hypothesis was tested using the χ^2 test. We obtained a value of $\chi^2 = 0.58$, which, with 1 degree of freedom and a significance level of 0.05, does not provide evidence to reject the independence hypothesis. Therefore, we conclude that our measurements are consistent with the absence of any correlation between consecutive homocoupling defects.

Table S3. Counts, relative frequencies, and probabilities for consecutive hh, ho/oh, and oo bonds, where "h" indicates a generic homocoupling bond and "o" a non-defective bond. The first two columns on the left contain the experimental values obtained from the STM images, while the two columns on the right are the expected counts and probability values (1)-(3). They were calculated assuming the hypothesis of independence between consecutive bonds and using the value $\Pr(\mathrm{h}) = 0.09$, as obtained from the measured relative frequency of homocoupling defects.

	Observed values		Expected values	
	# couples	relative frequency (%)	# couples	probability (%)
hh	7	0.6	9	0.8
ho / oh	185	17.0	182	16.7
oo	898	82.4	899	82.5
Total	1090	100.0	1090	100.0

6.1.2. Analysis of the density of defects as a function of polymer length

The second step was to evaluate whether shorter polymers exhibited a higher density of homocoupling defects, which could suggest that an increased occurrence of defects leads to premature termination of polymer growth. To investigate this, we examined the relationship between defect occurrence and polymer chain length by calculating the ratio of observed homocouplings to the total number of bonds between monomers. This ratio, representing the defect density, was analyzed as a function of polymer length.

Specifically, the polymers were categorized based on their length, defined as the number of bonds, n, that they comprise. The average number of defective couplings for polymers of length n was calculated using the expression:

$$\bar{D}_n = \frac{\sum_i^{N_n} D_{n,i}}{N_n} \tag{4}$$

where N_n represents the total number of polymers of length n that were analyzed, and $D_{n,i}$ denotes the number of defective couplings in the i-th polymer within this group. As a result, the average defect density for polymers of length n was determined as

$$\bar{\lambda}_n = \frac{\bar{D}_n}{n}. \tag{5}$$

The null hypothesis we aim to test in this instance is that there is no dependence of the average defect density on the polymer length, *i.e.* that the plot of $\bar{\lambda}_n$ as a function of n should appear as a horizontal line. The results reported in **Figure S18** support this hypothesis, showing that the measured values of $\bar{\lambda}_n$ are distributed in a seemingly random manner around, and relatively

close to, the value of 0.09, which corresponds to the overall frequency of defects observed in this polymer batch (see **Figure 2c**).

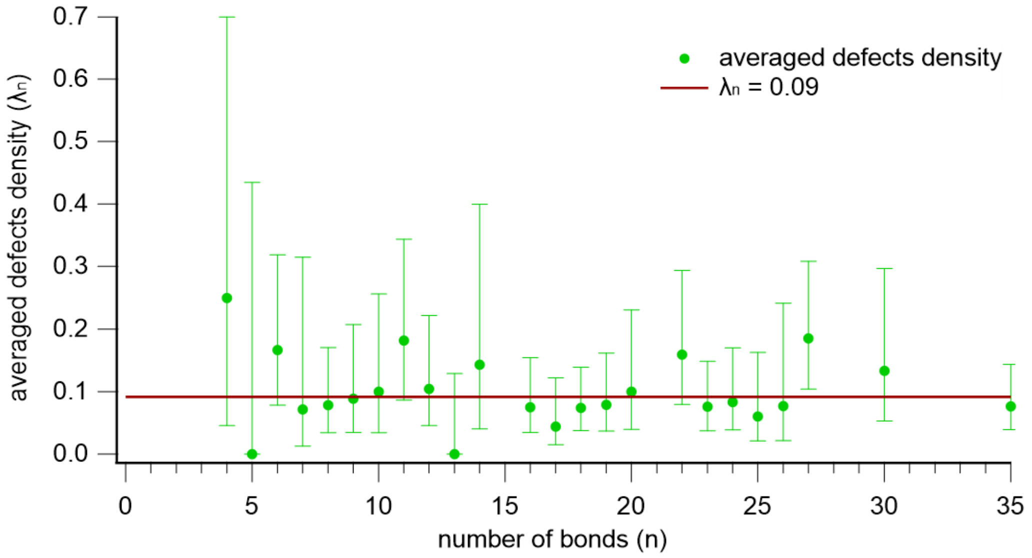

Figure S18. Average density of defects $\bar{\lambda}_n$ as a function of the polymer length, expressed by the number of bonds n. The uncertainties associated with each point are calculated as Wilson's score intervals with a confidence interval of 95% according to equation (6) and displayed as error bars. The continuous red horizontal line represents the overall average defect density value of 0.09.

However, a proper test of this hypothesis requires careful consideration of the uncertainties associated with each measured value of $\bar{\lambda}_n$. These uncertainties must account for both the spread of the individual $\lambda_{n,i}$ values and the varying number of polymers measured for different values of n. Since the $\bar{\lambda}_n$ values can be interpreted as success frequencies of binomial trials of length n,[18] the associated uncertainties can be evaluated using confidence intervals for binomial proportions.[18] Specifically, we opted to use Wilson's score interval,[19] which is particularly well-suited to this case as it is effective when sample sizes are small or when observed proportions are close to 0 or 1. In the present study, the sample size (N_n) was less than 10 for all values of n and, for some values of n, we observed $\bar{\lambda}_n$= 0.

The lower and upper bounds of Wilson's score interval (indicated in the following as $\bar{\lambda}_n^{min}$ and $\bar{\lambda}_n^{max}$, respectively) depend on the confidence interval, which we chose to be equal to 95%. In particular, these bounds are defined as:

$$\bar{\lambda}_n^{min/max} = \frac{\bar{\lambda}_n + \frac{z^2}{2B_n} \mp z \cdot \sqrt{\frac{\bar{\lambda}_n(1-\bar{\lambda}_n)}{B_n} + \frac{z^2}{4{B_n}^2}}}{1 + \frac{z^2}{B_n}} \quad (6)$$

where $B_n = n \cdot N_n$ is the total number of bonds measured across all polymers of length n (the total number of binomial trials of length n) and z is the critical value (or quantile) of a standard normal distribution.[19] For a 95% confidence interval, $z = 1.96$. The Wilson's score intervals for the different values of $\bar{\lambda}_n$ are indicated as error bars in **Figure S18**.

To test the hypothesis that $\bar{\lambda}_n$ is independent of n, we fitted a binomial generalized linear model (GLM) and tested the hypothesis that the slope of the line fitted through the $\bar{\lambda}_n$ values was zero. We note that the choice of a GLM instead of a linear regression test is dictated by the fact that the values of $\bar{\lambda}_n$ are bounded on both ends (they must be between 0 and 1) and that the number of defects follow binomial distributions.[20]

The estimated slope was –0.002 and the p-value for testing if this was significantly different from zero (p = 0.87). Therefore, there is no evidence to reject the null hypothesis and we can conclude that our measurements are consistent with the absence of any dependence of the defect density on the polymer length.

6.1.3. Analysis of the density of defects at the polymer ends

Finally, we aimed to determine whether defects preferentially accumulated at the ends of polymers, irrespectively of their length. Such a pattern could in fact support a potential correlation between the presence of defects and the termination of the polymerization process.

To conduct this analysis, we classified each bond within a polymer into one of two categories: "ending bonds" (bonds connecting the first or last monomer to its neighbor) and "central bonds" (all other bonds). For polymers whose ends were fully visible within the STM images, this classification was straightforward. However, for polymers extending beyond the image boundaries, we considered only the bonds forming the central portion of the backbone and excluded those closest to the edges of the image. This approach ensured that terminal bonds at the image borders were not erroneously categorized as central bonds. The results of this classification, including the absolute number of defects and the relative defect frequency f observed in central and ending bonds, are presented in **Table S4**.

Table S4. Statistics of the number of defective bonds in the center and at the ends of the polymer chains. Defects in the center are counted across all bonds, excluding the first and last bonds of each sequence. Defects at the ends are counted over all visible final bonds in the images. The corresponding uncertainties (expressed as uncertainty intervals) are calculated as Wilson's score intervals according to equation (7).

	Central bonds 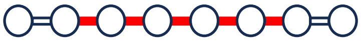	Ending bonds 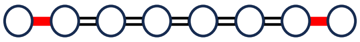
Total number of bonds ***B***	1014	53
Total number of defects ***D***	93	7
Defect frequency ***f*** (%)	9.2	13.2
Uncertainty interval (%)	[7.6 ; 11.0]	[6.5 ; 24.9]

If the termination of polymer growth were positively influenced by the presence of defects, one would expect polymer defects to be more likely at the ends of the polymer chains than in the middle. The null hypothesis, therefore, is that the defect frequency of ending bonds is equal to that of central bonds. As in the case discussed in the previous section, testing this hypothesis also requires evaluating the uncertainties associated with the experimentally measured defect frequencies. In particular, since the number of ending bonds is significantly lower than the number of central bonds, the uncertainty in the defect frequency for ending bonds is expected to be higher.

Similar to the previous case, these uncertainties can be evaluated using Wilson's score interval method. This approach is appropriate because, like the defect densities $\bar{\lambda}_n$, the defect frequencies f can also be interpreted as success frequencies of binomial trials. This is particularly relevant for ending bonds, where the corresponding sample size is small. Accordingly, the upper and lower bounds, $f^{min/max}$, can be expressed as:

$$f^{min/max} = \frac{f + \frac{z^2}{2B} \pm z \cdot \sqrt{\frac{f(1-f)}{B} + \frac{z^2}{4B^2}}}{1 + \frac{z^2}{B}} \qquad (7)$$

where, for each of the two classes of bonds, B is the total number of measured bonds, D is the total number of defective bonds, $f = D/B$, and z = 1.96 is the critical value from the standard normal distribution for a 95% confidence level. The resulting intervals are reported as uncertainty intervals in **Table S4**.

To test the null hypothesis, we conducted both a χ^2 test and a Fisher's exact test. The latter was included as an additional precaution due to the small sample sizes in some of the counts. The

χ^2 test resulted in a value of χ^2 = 0.41 on 1 degree of freedom (using Yates's continuity correction), with a corresponding p-value of p = 0.52. The p-value for Fisher's exact test was p = 0.35. Considering a significance level of 0.05, for both tests we accept the null hypothesis and conclude that our measurements are consistent with the absence of any accumulation of defects at the ends of the polymer chains.

In summary, the detailed statistical analysis enabled by the precise ESD-STM sequencing of the polymers suggests that the shorter chain length of conventional pgBTTT compared to homocoupling-free pgBTTT cannot be directly attributed to the presence of homocoupling defects.

6.2. Polymer 2D assembly and side chain interdigitation patterns

In both conventional and homocoupling-free pgBTTT, we observed two main periodic patterns formed by the ordered interdigitation of the side chains, as shown in **Figure S19**, where these patterns are highlighted in different colors. These configurations, also depicted in **Figure S20**, correspond to two distinct assembly arrangements of the side chains, as previously proposed,[21] where the polymer backbones are defect-free. Indeed, our fitted images reveal that these regular patterns were consistently associated with non-defective backbone segments in both batches. While the absence of homocoupling defects is a necessary condition for the formation of these ordered patterns, it is not sufficient. In fact, we also observed various disordered areas, even in homocoupling-free pgBTTT, due to factors such as differing degrees of local surface coverage, polymer crossings, impurities, and backbone kinks. This is particularly significant for homocoupling-free pgBTTT, where the very long backbones tend to get entangled with each other, strongly affecting the assembly on the surface.

Nevertheless, the observation that regular assembly patterns correspond to defect-free backbones suggests that extended regions with this type of packing are more likely to form in homocoupling-free pgBTTT compared to the conventional one.

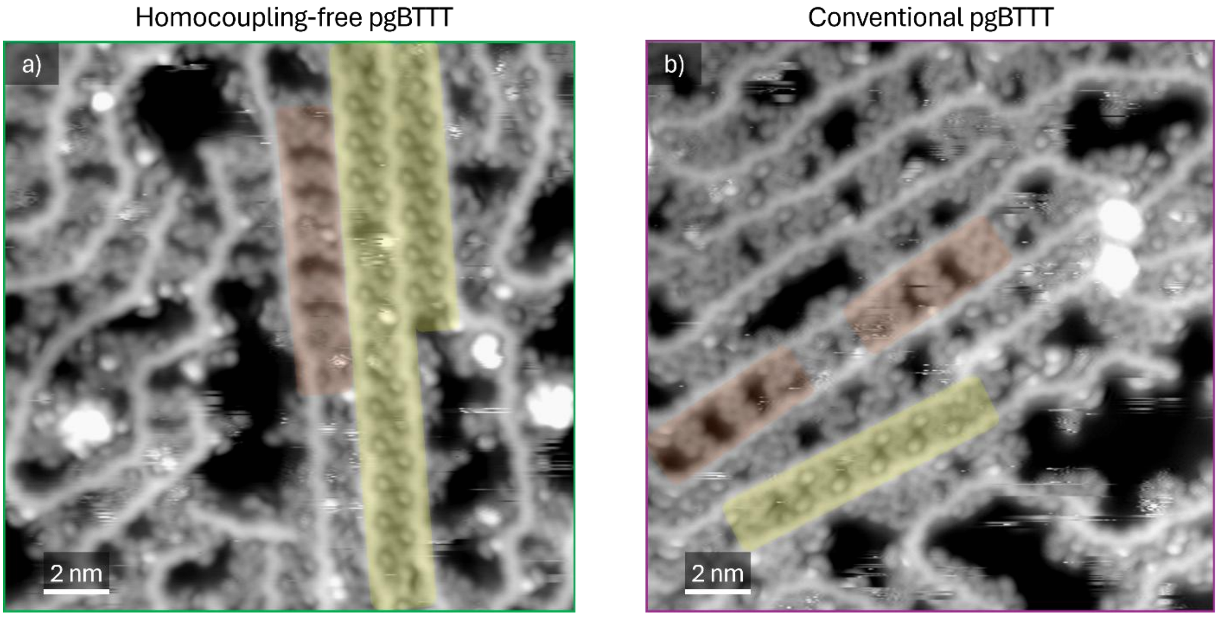

Figure S19. STM images of the 2D assembly of homocoupling-free a) and conventional b) pgBTTT. Both polymers show two types of regular interdigitation patterns of the side chains, highlighted in orange and yellow, respectively.

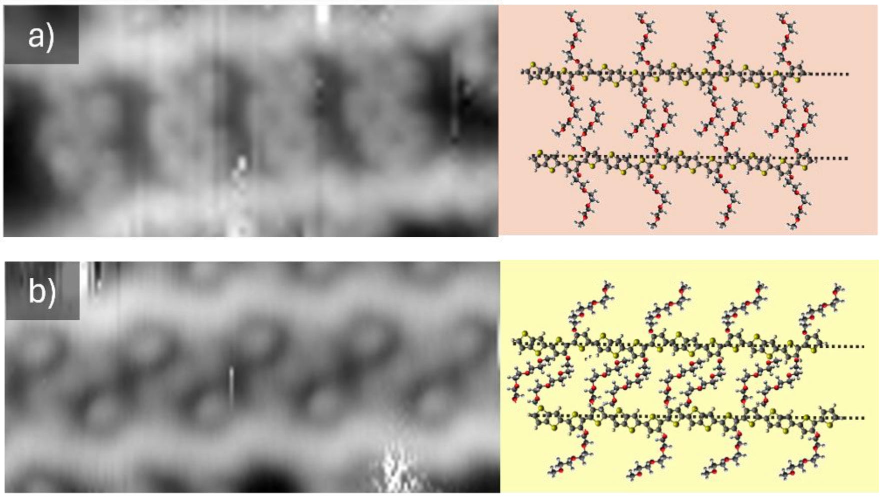

Figure S20. Zoom-in on the two interdigitation patterns observed for the two batches of pgBTTT, revealing differences in how the glycolated side chains orient and interdigitate with neighboring chains. In pattern a) (orange), the side chains form a larger angle with the backbone and are slightly less densely packed compared to the pattern in b) (yellow), resulting in a slightly greater distance between adjacent backbones (2D lamellar separation). Details about all the possible molecular configurations that can be associated to these patterns are discussed in previous work.[21]

6.3. Analysis of the mass distributions

To obtain the experimental mass distributions shown in **Figure 3**, the polymers from each batch (homocoupling-free and conventional pgBTTT) were divided into two groups. The first group includes polymers that are fully contained within the boundaries of the STM images, with their profiles visible from start to end. These are referred to as "full" polymers. The corresponding mass distributions for homocoupling-free pgBTTT and conventional pgBTTT are displayed in **Figure S21a** and **S21b**, respectively. However, considering only the "full" polymers would introduce a bias toward shorter chains, as longer polymers are more likely to extend beyond the edges of the STM image or to overlap with other polymers, making their exact length challenging to determine unambiguously. To address this issue, we employ survival analysis, a statistical method that accounts for polymers partially contained within STM images or intersecting with other polymers (referred to as "longer than" polymers). This method uses the distribution of the partially visible polymers to correct the mass distribution of the "full" polymers. A detailed explanation of how survival analysis can be applied to determine the mass distribution of conjugated polymers, along with benchmarking against NMR and GPC, and its ability to overcome the limitations of these traditional techniques, is provided in previous work.[22] Here, we emphasize that while our previous work primarily focused on polymers with relatively low DP_n, such as the conventional Stille batch studied here, the analysis of homocoupling-free pgBTTT required additional consideration due to the significantly greater length of its polymer strands. Longer polymers have a higher likelihood of crossing the edge of STM images or becoming entangled with other polymers. To accommodate this, neighboring areas were imaged by STM and the images stitched together to enable complete tracing of these extremely long polymers.

Figure S21c and **S21d** show the mass distributions of the "longer than" polymers, while the results of the survival analysis correction are shown in **Figure 3** and in **Figures S21a** and **S21b**, overlaid onto the original "full" polymer data. Consistent with previous results,[22] the corrected distributions can be modelled as the product of a Flory-Schulz geometric distribution with a logistic function. The latter accounts for the Soxhlet extraction process used to purify the sample from shorter oligomers, unreacted monomers, and residual catalyst. Thus, the probability function for a polymer having length x can be described as:

$$\Pr(x) = C\frac{1}{1+e^{-k(x-x_0)}}p^{x-1}(1-p) \qquad (8)$$

where C is a normalizing constant, k is the steepness of the logistic curve, x_0 is its center, and p is the extent of reaction in the Flory–Schulz distribution.[22]

The individual contributions of the Flory-Schulz distribution and the logistic function for both homocoupling-free and conventional Stille pgBTTT are shown in **Figure S21e** and **S21f**, respectively, alongside the corresponding k, x_0, and p parameters. A comparison of these parameters between the two polymer batches highlights the differences in their synthetic routes, particularly concerning the purification process. Conventional pgBTTT was subjected to Soxhlet extractions in methanol, acetone, hexane, THF, and chloroform, consecutively, following the reported purification method for original pgBTTT.[8] However, when this solvent sequence was applied to purify homocoupling-free pgBTTT, it was observed that little to no material eluted in the acetone, hexane, and THF fractions. This was attributed to the symmetric Stille synthesis, which likely produced more chains with higher molar mass, limiting their solubility and therefore elution in these solvents. To address the limited separation of polymer chains, DCM was introduced as an additional Soxhlet solvent prior to chloroform. This modification allowed for further separation of the higher molar mass fraction of homocoupling-free pgBTTT and is reflected in a correspondingly higher mass onset and a less steep filtering of the logistic function (see **Figure S21e** and **Figure S21f** for comparison).

However, it must be noted that the p values for the two polymer batches also differ significantly, with the symmetric Stille polymerization yielding p = 0.96 ± 0.01 and the standard Stille polymerization yielding p = 0.92 ± 0.01. This difference is reflected in the longer polymer chains produced by the former method compared to the latter and is further corroborated by the higher DP_n and M_n values (see **Figure 3**).

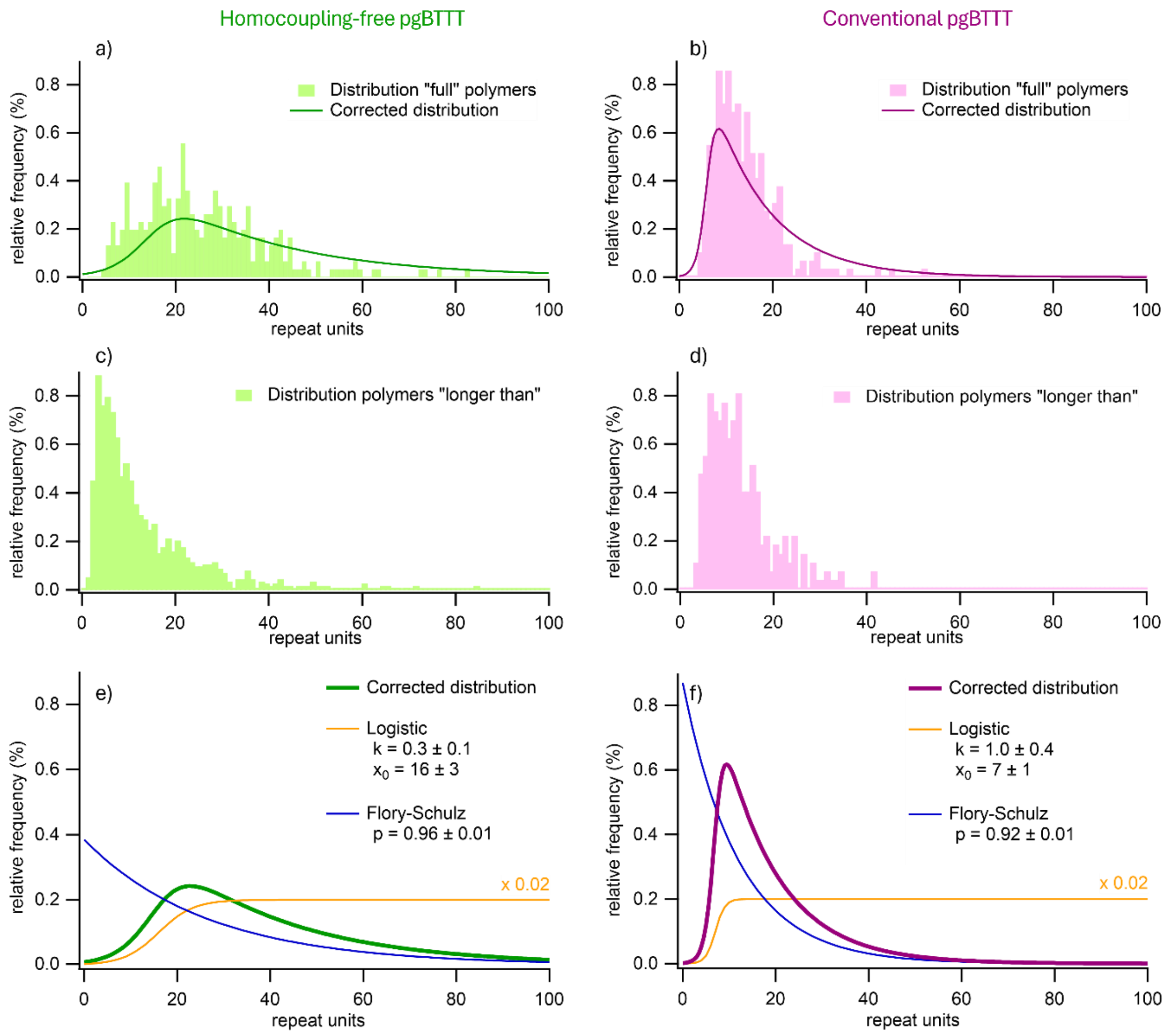

Figure S21. ESD-STM mass distributions of "full" polymers for a) homocoupling-free and b) conventional Stille pgBTTT. These distributions were corrected using the survival analysis method, by using the distributions of "longer than" polymers shown in c) for homocoupling-free and d) for conventional Stille pgBTTT. The resulting corrected distributions are displayed as continuous lines in a) and b), respectively. For clearer comparison, the data are displayed as relative frequencies, since different numbers of profiles were collected: 305 polymers in a), 291 in b), 1130 in c), and 272 in d). The individual contributions of the Flory-Schulz and the logistic components to the corrected distributions are also shown in e) and f), along with the corresponding parameters derived from the survival analysis.

7. ICP-MS

Since it has been reported that Pd residues can negatively impact OECT performance, both conventional and homocoupling-free pgBTTT were scavenged post-polymerization with diethylammonium diethyldithiocarbamate. It was hypothesized by Griggs *et al*.[23] that residual Pd may function as a charge carrier trap or co-catalyst for the oxygen reduction reaction, thereby reducing the stability of the channel material. Consequently, minimizing the Pd concentration in OECT materials is imperative. The resulting Pd concentrations were quantified using ICP-MS and are reported in **Table S5**. All polymers exhibited low Pd concentrations, demonstrating the effectiveness of the scavenging process and attesting to their comparability when used in OECTs. Additionally, the Sn and Fe content of the polymers was assessed as well.

Table S5. ICP-MS results for regular and homocoupling-free pgBTTT.

pgBTTT	Pd (ppm)	Sn (ppm)	Fe (ppm)
Conventional	62.1	45.8	103.6
Hc-free: batch 1	64.7	240.6	160.0
Hc-free: batch 2	9	12	73
Hc-free: batch 3	148	78	BDL[a)]

[a)] BDL = below detection limit

8. OECTs

It is important to note that the OECT results reported here are derived in the linear regime, as opposed to the standard saturation operating regime. The reasoning behind this is to keep the device in the low carrier density regime, where polarons induce structural ordering, as opposed to pushing them to high carrier densities, where charge-charge interactions become significant and irreversible structural disordering occurs.[24] The standard expression for g_m derived from the Bernards-Malliaras model is formally valid only in the saturation regime.[25] However, Bernards and Malliaras also derived an expression for the drain current in the linear regime:[26]

$$I_D = G\left[1 - \frac{V_G - \frac{1}{2}V_D}{V_p}\right]V_D \qquad (9)$$

where G is the channel conductance and V_p is the pinch-off voltage, which corresponds to the onset of the saturation region.

Equation (9) is the OECT analogue of the linear regime in MOSFETs, typically expressed as:[27]

$$I_D = \frac{W}{L}\mu_{eff}C_{ox}\left(V_G - V_{TH} - \frac{1}{2}V_D\right)V_D \qquad (10)$$

with μ_{eff} the effective mobility, C_{ox} the specific gate-dielectric capacitance per unit area, and V_{TH} the threshold voltage, which is analogues to V_p in the Bernards-Malliaras model.

The transconductance is then obtained as the derivative of the transfer curve:

$$g_m = \frac{\partial I_D}{\partial V_G} = -\mathrm{G}\frac{V_D}{V_p} \qquad (11)$$

Here, $G = q\mu p_o WT/L$ and $V_p = qp_o\mathrm{T}/c_d$, where q is the elementary charge, μ the mobility, p_o the initial hole density in the semiconductor before application of a gate voltage, W the channel width, T the thickness, L the length and c_d the capacitance per unit area. Substituting these definitions into Equation (11) yields:

$$g_m = -\frac{W}{L}\mu c_d V_D \qquad (12)$$

Rewriting the capacitance term (c_d) in terms of the volumetric capacitance (C^*) gives:

$$g_m = -\frac{WT}{L}\mu C^* V_D \qquad (13)$$

Equation (13) provides a route to extract μC^* from OECTs operated in the linear regime.[28] In principle, the extracted values should be comparable to those obtained in saturation. However, because both μ and C^* are voltage-dependent, values determined in the saturation regime are often slightly higher.[29]

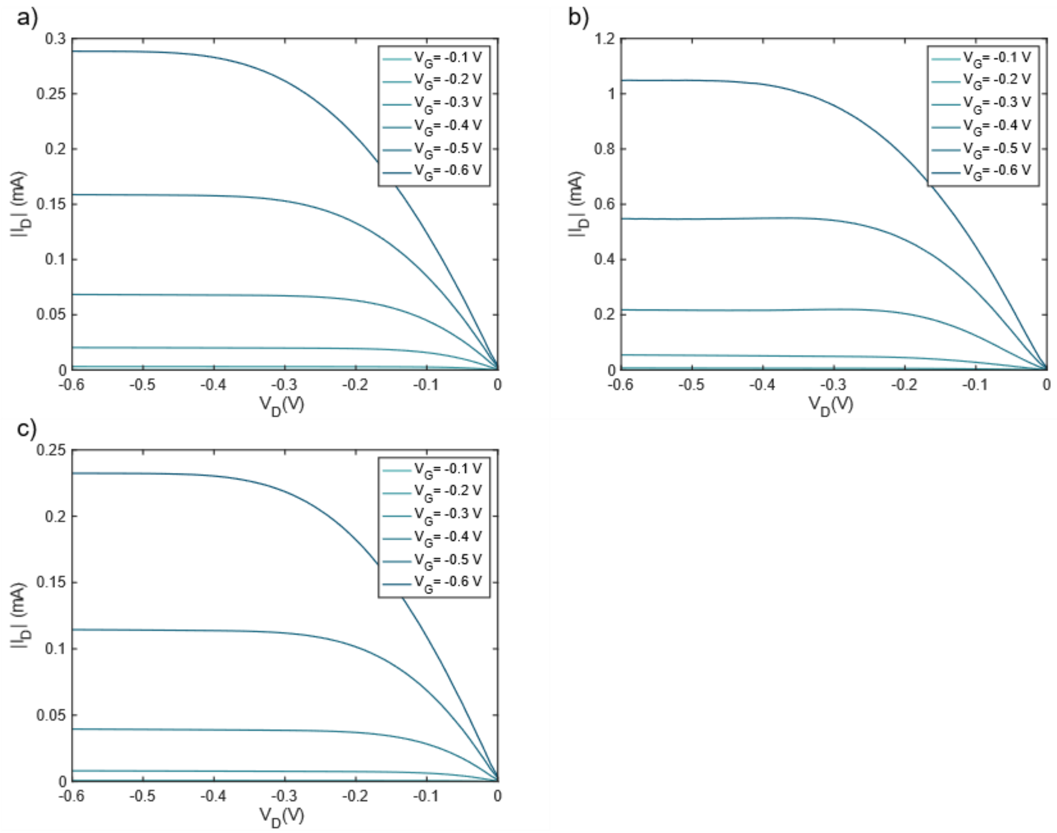

Figure S22. Organic electrochemical transistor output curves (I_D vs. V_D) for the devices made from conventional pgBTTT (a), homocoupling-free pgBTTT (b), and homocoupling-free pgBTTT from the DCM fraction (c). All curves were obtained at a sweep rate of 100 mV s^{-1} in nitrogen environment.

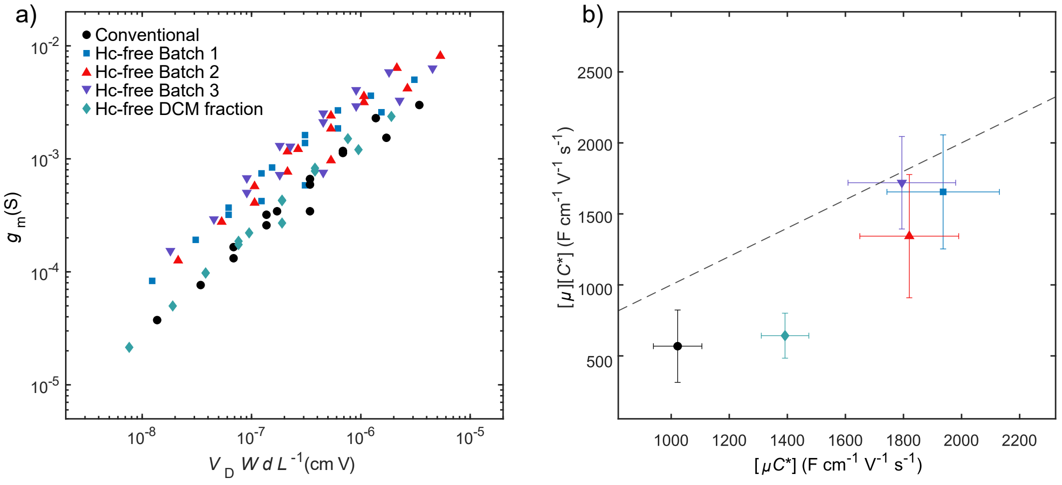

Figure S23. Comparison of the μC^* values for the three batches of homocoupling-free pgBTTT, conventional pgBTTT, and the DCM fraction of homocoupling-free pgBTTT: a) Transconductance (g_m) as a function of the drain voltages and geometry of the channels. Each point represents one measurement. The measurement was conducted in the linear region with various drain voltages and channel lengths. b) The product of the μ and C^* values measured individually as a function of [μC^*] values computed from the linear slopes of the data in (a). The dashed line represents a 1:1 agreement between the values. Error bars represent the root-mean-square error of the values.

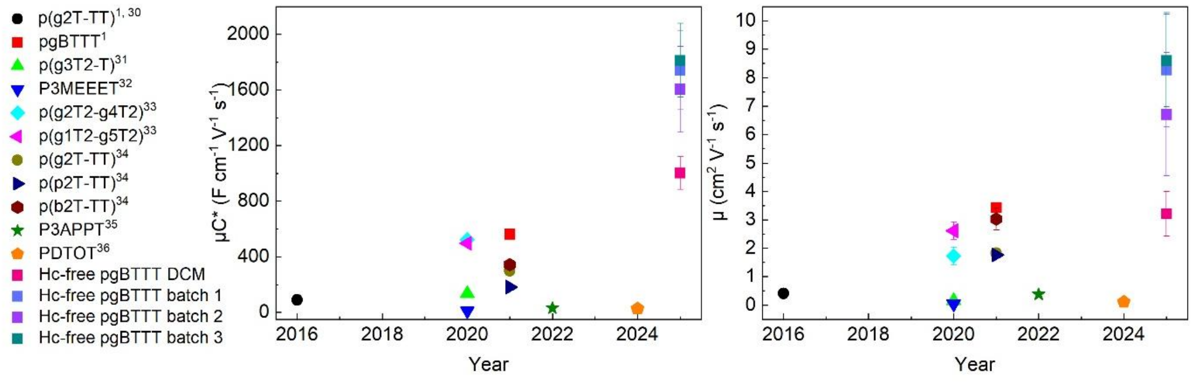

Figure S24. Comparison of the μC^* and μ values of homocoupling-free pgBTTT to previously reported thiophene-based enhancement-mode p-type materials.[8,30-36] For all homocoupling-free pgBTTT batches, the average of the μC^* and μ x C^* values, as reported in **Table 1**, is shown.

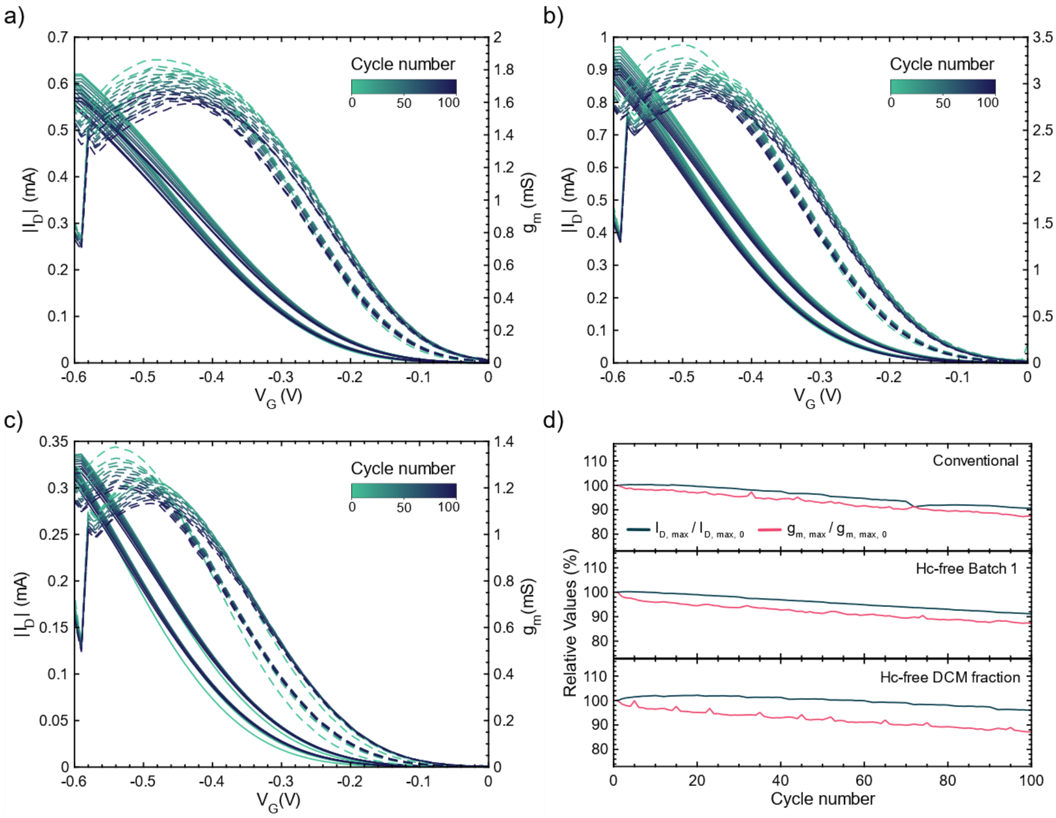

Figure S25. Operational stability of pgBTTT OECT devices made of conventional pgBTTT (a), homocoupling-free pgBTTT (b), and homocoupling-free pgBTTT from the DCM fraction (c). The solid lines represent the drain current, I_D (left axis), and the dashed lines represent the transconductance, g_m (right axis). The channel dimensions of the devices are 50 µm in length and 100 µm in width. Each OECT device was cycled 100 times with a gate voltage V_G from 0 to −0.6 V at a drain voltage $V_D = -0.1$ V with a scan rate of 100 mV s^{-1}. Data were plotted for the 1st and each 10th cycle. d) Relative values of the maximum I_D and maximum g_m calculated as a percentage of the maximum I_D and g_m of the first cycle, respectively.

9. Mobility measurements

The hole mobilities, μ, of the polymers were estimated by measuring the charge carrier transit time, τ_e, in OECT devices (**Fig. S26a**). By controlling the gate current instead of the gate bias, it is possible to control the number of charges injected into the channel and thus deduce the charge carrier mobilities independently of the channel capacitance, C^*. This method relies on the transient Bernards and Malliaras model, where the drain current I_D is described as a function of gate current I_G and time t,

$$I_D(t, I_G) = I_O - I_G(f + \frac{t}{\tau_e}) \tag{14}$$

where I_O is the drain current without the gate current, and f is the weighting factor that depends on the drain and gate bias.[26] By taking the derivative of the above expression with respect to time, we can express I_D only as a function of I_G and τ_e.

$$\frac{dI_D}{dt} = -\frac{I_G}{\tau_e} \tag{15}$$

By computing the slope of the dI_D/dt versus I_G curve (**Fig. S26b**), we can estimate the inverse of τ_e, which is defined as $\tau_e = L^2/\mu V_D$. The charge carrier transit time was calculated for the two OECT devices with the largest channel dimensions (L = 500 µm, 200 µm and 100 µm) to allow sufficient charge to enter the channel and obtain a reasonable number of data points. The table of collected transit time and mobility values is added below (**Table S6**).

Table S6. Estimated charge carrier transit time and hole mobility values for the conventional and homocoupling-free (Hc-free) pgBTTT batches. All devices have a width of W = 100 µm.

	Conventional					Hc-free DCM fraction				
L (µm)	100	200	100	200	500	100	200	100	200	500
τ_e (µs)	2520	388	1920	880	188	2260	687	3150	895	173
μ (cm^2 V^{-1}s^{-1})	2.52	1.55	1.92	3.52	4.69	2.26	2.75	3.15	3.58	4.32
	Hc-free Batch 1					Hc-free Batch 2				
L (µm)	100	200	100	200	500	100	200	100	200	500
τ_e (µs)	6230	1970	6600	2480	433	6890	945	6940	2460	245
μ (cm^2 V^{-1}s^{-1})	6.23	7.88	6.60	9.83	10.8	6.89	3.78	6.94	9.84	6.13
	Hc-free Batch 3									
L (µm)	100	200	100	200	500					
τ_e (µs)	6740	2280	6990	2510	403					
μ (cm^2 V^{-1}s^{-1})	6.74	9.14	6.99	10.0	10.1					

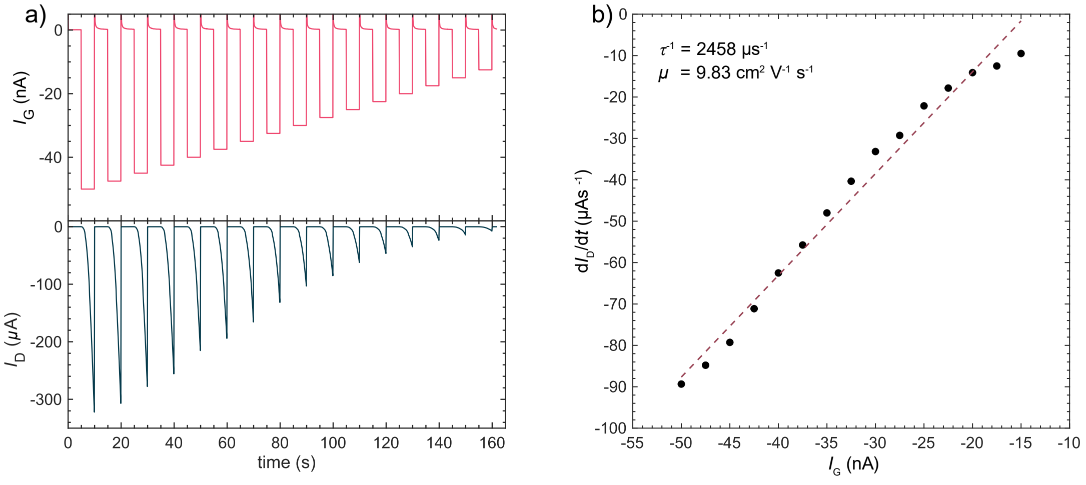

Figure S26. Charge carrier transit time (τ_e) and hole mobility (μ) estimation for hc-free pgBTTT (batch 1) by pulsing the OECT device with constant gate current values at V_D = –0.1 V. a) Pulsed gate current (I_G) and the corresponding drain current response. The gate current pulses were applied for 5 s, with 5 s rest in between. dI_D/dt was collected by taking the derivative of the I_D response with respect to time. Different values for I_G were used to extract various dI_D/dt values. b) dI_D/dt values collected from the measurement in (a). The slope extracted was used to compute τ_e and therefore μ.

10. Electrochemical impedance spectroscopy

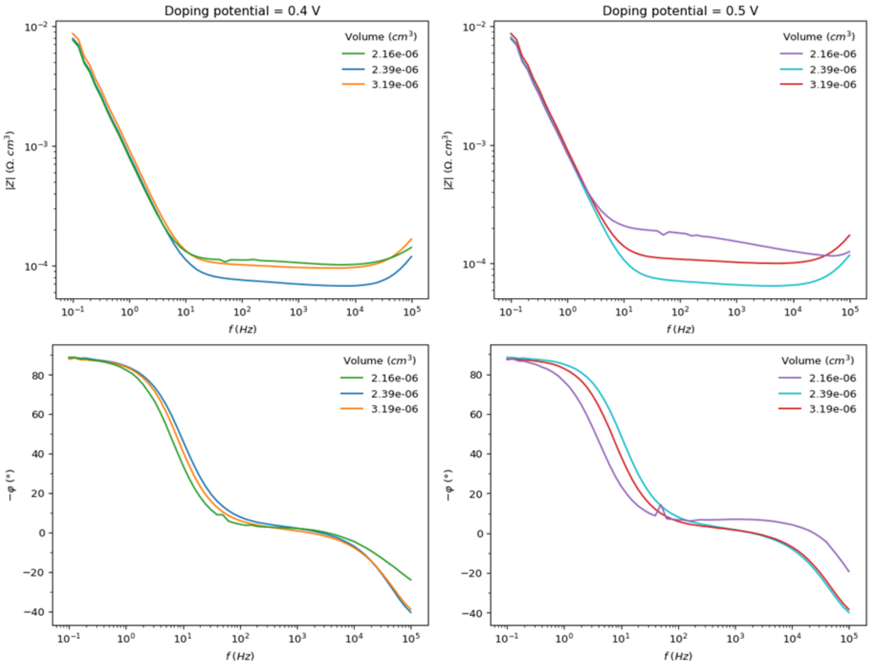

Figure S27. Electrochemical impedance spectroscopy (Bode plots) for three conventional pgBTTT devices at doping potentials of 0.4 (left) and 0.5 V (right). The impedance (top) is normalized by the film volume.

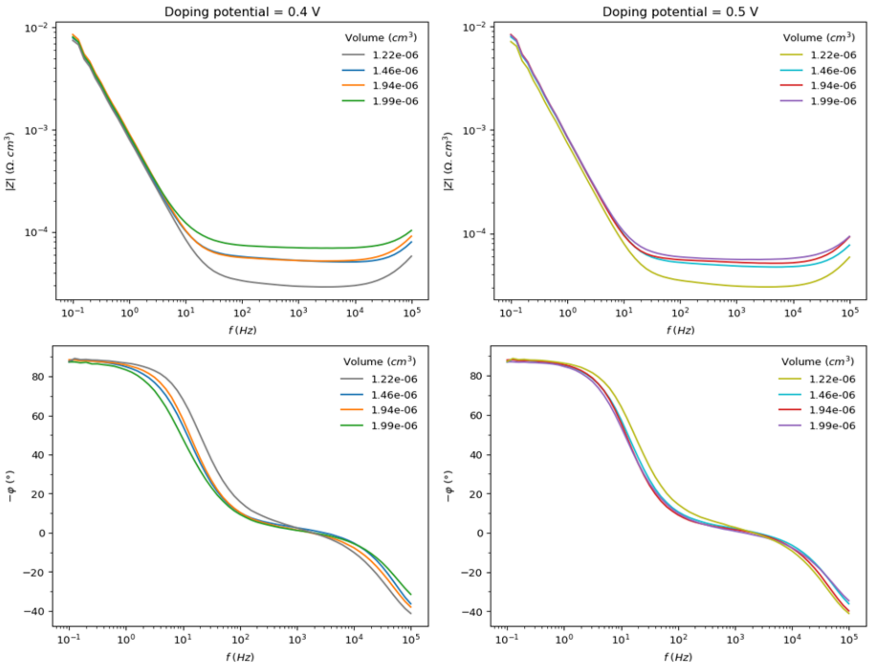

Figure S28. Electrochemical impedance spectroscopy (Bode plots) for four lower-molar-mass homocoupling-free pgBTTT (DCM fraction) devices at doping potentials of 0.4 (left) and 0.5 V (right). The impedance (top) is normalized by the film volume.

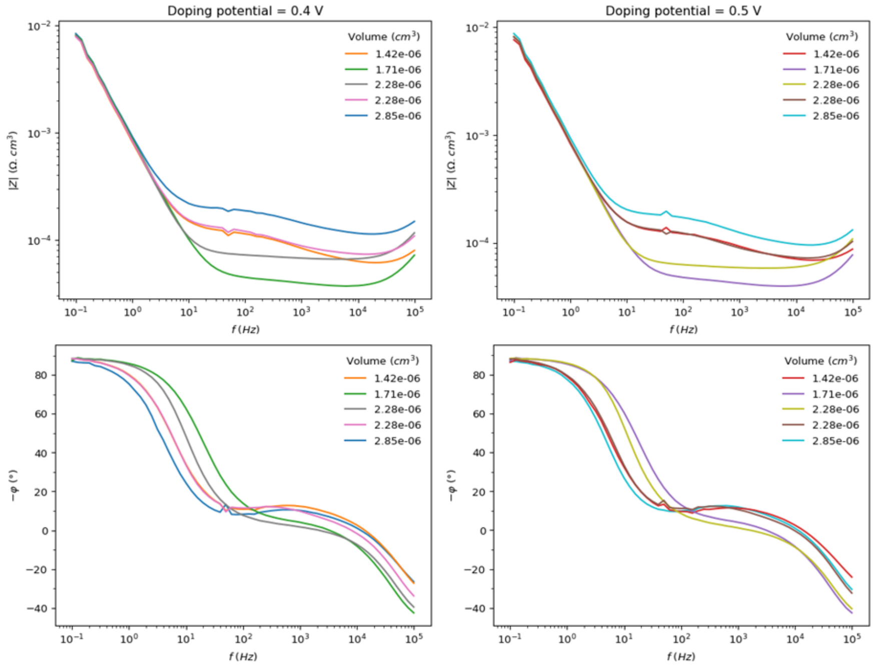

Figure S29. Electrochemical impedance spectroscopy (Bode plots) for five higher-molar-mass homocoupling-free pgBTTT (chloroform fraction; batch 1) devices at doping potentials of 0.4 (left) and 0.5 V (right). The impedance (top) is normalized by the film volume.

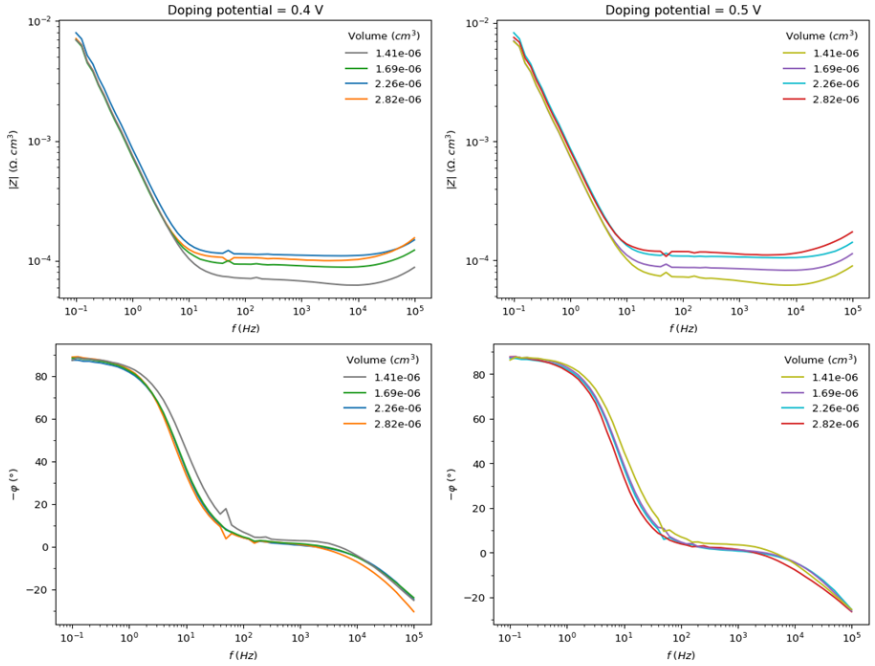

Figure S30. Electrochemical impedance spectroscopy (Bode plots) for four homocoupling-free pgBTTT (batch 2) devices at doping potentials of 0.4 (left) and 0.5 V (right). The impedance (top) is normalized by the film volume.

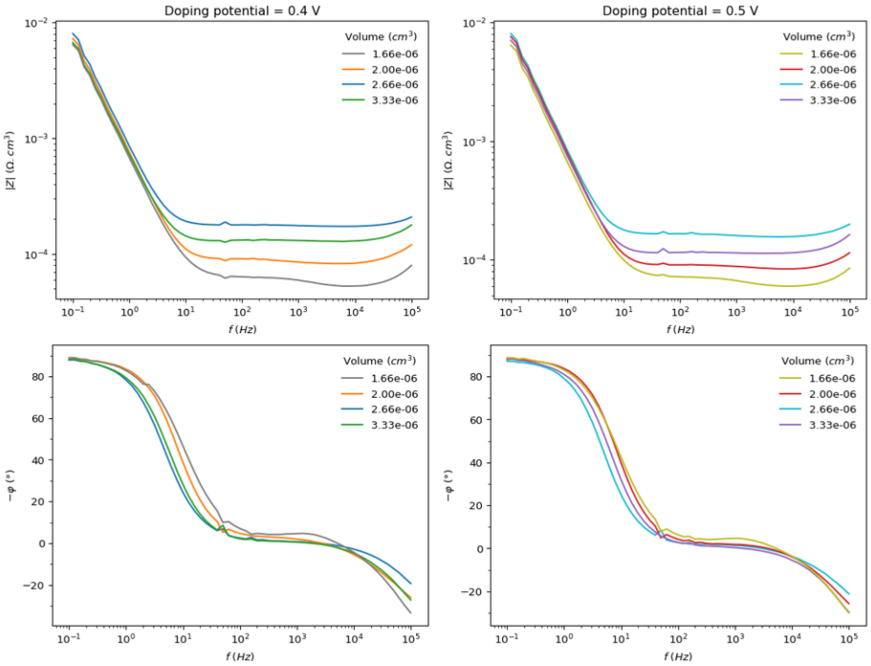

Figure S31. Electrochemical impedance spectroscopy (Bode plots) for four homocoupling-free pgBTTT (batch 3) devices at doping potentials of 0.4 (left) and 0.5 V (right). The impedance (top) is normalized by the film volume.

11. GIWAXS

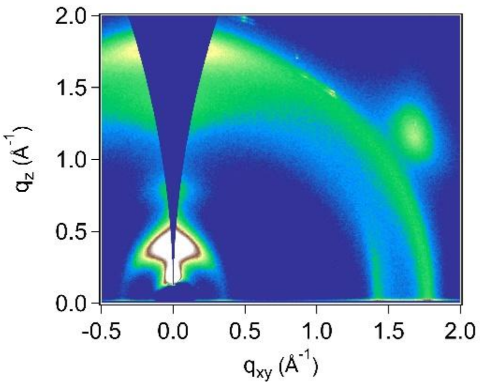

Figure S32. Two-dimensional GIWAXS pattern for lower-molar-mass (DCM fraction) homocoupling-free pgBTTT.

Table S7. Overview of the observed 2D GIWAXS peaks for the conventional and homocoupling-free (hc-free) pgBTTT batches.

Index		Q_z (out-of-plane) Conventional	Hc-free	Hc-free DCM	Q_{xy} (in-plane) Conventional	Hc-free	Hc-free DCM
	q (Å^{-1})	0.42	0.42	0.39	0.38	0.38	0.37
(100)	D (Å)	14.97	14.97	16.11	16.53	16.53	16.98
	q (Å^{-1})	0.86	0.86	0.79	-	-	-
(200)	D (Å)	-	-	-	-	-	-
	q (Å^{-1})	1.75	1.72	1.77	1.75	1.70	1.77
(010)	D (Å)	3.59	3.65	3.54	3.59	3.70	3.55
	q (Å^{-1})	-	-	-	1.44	1.42	1.43
(001)	D (Å)	-	-	-	4.36	4.42	4.39
	q (Å^{-1})	1.32	1.30	-	-	-	-
broad signal	D (Å)	4.76	4.83	-	-	-	-

Table S8. Estimation of the coherence length according to the Scherrer equation, $L_c = 2\pi K/q$, with shape factor K = 0.9.[37]

Index		Q_z (out-of-plane) Conventional	Hc-free	Q_{xy} (in-plane) Conventional	Hc-free	Hc-free DCM
(100)	L_c (Å)	98.7	52.7			
(010)	L_c (Å)			45.8	46.9	57.3
(001)	L_c (Å)			50.8	98.7	

The (001) reflection appears sharper, more pronounced and with a higher coherence length in homocoupling-free pgBTTT compared to conventional pgBTTT (**Table S8**). This signal was originally debated by Hallani *et al.* to originate either from a n+2 order reflection of the

repeating unit length (13.3 Å) or the spacing between the triethylene glycol side chains.[8,38] This sharper signal was also observed in the homocoupling-free alkoxylated PBTTT-$(OR)_2$ derivative.[12] If the signal was indeed to be attributed to the repeating unit length, it would be expected to be significantly more prominent in the case of the homocoupling-free variant and would likely result in the observation of higher order signals, similar to those seen in pBTTT. Conversely, in view of the homocoupling-free material always consisting of the correct repeating unit, the spacing between the side chains is expected to be more consistent throughout the whole material and therefore interdigitation of the side chains is expected to be generally improved throughout the crystallites, resulting in a sharper signal. Qualitatively, this is in line with the STM data (**Figure 2 and S19**), where the side chain packing was observed to be more consistent. We thus conclude that the (001) reflection is likely to represent the regular spacing between the triethylene glycol side chains.

12. References

(1) Horcas, I.; Fernandez, R.; Gomez-Rodriguez, J. M.; Colchero, J.; Gomez-Herrero, J.; Baro, A. M., WSXM: a software for scanning probe microscopy and a tool for nanotechnology. *Rev. Sci. Instrum.* **2007**, *78* (1), 013705. DOI: 10.1063/1.2432410

(2) Nečas, D.; Klapetek, P., Gwyddion: an open-source software for SPM data analysis. *Open Physics* **2012**, *10* (1), 181-188. DOI: 10.2478/s11534-011-0096-2

(3) Hanwell, M. D.; Curtis, D. E.; Lonie, D. C.; Vandermeersch, T.; Zurek, E.; Hutchison, G. R., Avogadro: an advanced semantic chemical editor, visualization, and analysis platform. *J. Cheminform.* **2012**, *4* (1), 17. DOI: 10.1186/1758-2946-4-17

(4) LMAPper - The SPM and Mol Viewer | Reviews for LMAPper - The SPM and Mol Viewer at SourceForge.net. https://sourceforge.net/projects/spm-and-mol-viewer/reviews/

(5) Ilavsky, J., Nika: software for two-dimensional data reduction. *J. Appl. Crystallogr.* **2012**, *45* (2), 324-328. DOI: 10.1107/S0021889812004037

(6) Oosterhout, S. D.; Savikhin, V.; Zhang, J.; Zhang, Y.; Burgers, M. A.; Marder, S. R.; Bazan, G. C.; Toney, M. F., Mixing Behavior in Small Molecule:Fullerene Organic Photovoltaics. *Chem. Mater.* **2017**, *29* (7), 3062-3069. DOI: 10.1021/acs.chemmater.7b00067

(7) Murbach, M.; Gerwe, B.; Dawson-Elli, N.; Tsui, L.-k., impedance.py: A Python package for electrochemical impedance analysis. *J. Open Source Softw.* **2020**, *5* (52), 2349. DOI: 10.21105/joss.02349

(8) Hallani, R. K.; Paulsen, B. D.; Petty, A. J., 2nd; Sheelamanthula, R.; Moser, M.; Thorley, K. J.; Sohn, W.; Rashid, R. B.; Savva, A.; Moro, S.; Parker, J. P.; Drury, O.; Alsufyani, M.; Neophytou, M.; Kosco, J.; Inal, S.; Costantini, G.; Rivnay, J.; McCulloch, I., Regiochemistry-Driven Organic Electrochemical Transistor Performance Enhancement in Ethylene Glycol-Functionalized Polythiophenes. *J. Am. Chem. Soc.* **2021**, *143* (29), 11007-11018. DOI: 10.1021/jacs.1c03516

(9) Madathil, P. K.; Cho, S.; Choi, S.; Kim, T.-D.; Lee, K.-S., Synthesis and Characterization of Cyclopentadithiophene and Thienothiophene-Based Polymers for Organic Thin-Film Transistors and Solar Cells. *Macromol. Res.* **2018**, *26* (10), 934-941. DOI: 10.1007/s13233-018-6130-0

(10) Rudenko, A. E.; Thompson, B. C., Optimization of direct arylation polymerization (DArP) through the identification and control of defects in polymer structure. *J. Polym. Sci., Part A: Polym. Chem.* **2015**, *53* (2), 135-147. DOI: 10.1002/pola.27279

(11) Ma, B.; Shi, Q.; Ma, X.; Li, Y.; Chen, H.; Wen, K.; Zhao, R.; Zhang, F.; Lin, Y.; Wang, Z.; Huang, H., Defect-Free Alternating Conjugated Polymers Enabled by Room-Temperature Stille Polymerization. *Angew. Chem. Int. Ed. Engl.* **2022**, *61* (16), e202115969. DOI: 10.1002/anie.202115969

(12) Vanderspikken, J.; Liu, Z.; Wu, X. C.; Beckers, O.; Moro, S.; Quill, T. J.; Liu, Q.; Goossens, A.; Marks, A.; Weaver, K.; Hamid, M.; Goderis, B.; Nies, E.; Lemaur, V.;

Beljonne, D.; Salleo, A.; Lutsen, L.; Vandewal, K.; Van Mele, B.; Costantini, G.; Van den Brande, N.; Maes, W., On the Importance of Chemical Precision in Organic Electronics: Fullerene Intercalation in Perfectly Alternating Conjugated Polymers. *Adv. Funct. Mater.* **2023**, *33* (52), 2309403. DOI: 10.1002/adfm.202309403

(13) Sahalianov, I.; Hynynen, J.; Barlow, S.; Marder, S. R.; Muller, C.; Zozoulenko, I., UV-to-IR Absorption of Molecularly p-Doped Polythiophenes with Alkyl and Oligoether Side Chains: Experiment and Interpretation Based on Density Functional Theory. *J. Phys. Chem. B* **2020**, *124* (49), 11280-11293. DOI: 10.1021/acs.jpcb.0c08757

(14) Jacobs, I. E.; Lin, Y.; Huang, Y.; Ren, X.; Simatos, D.; Chen, C.; Tjhe, D.; Statz, M.; Lai, L.; Finn, P. A.; Neal, W. G.; D'Avino, G.; Lemaur, V.; Fratini, S.; Beljonne, D.; Strzalka, J.; Nielsen, C. B.; Barlow, S.; Marder, S. R.; McCulloch, I.; Sirringhaus, H., High-Efficiency Ion-Exchange Doping of Conducting Polymers. *Adv. Mater.* **2022**, *34* (22), e2102988. DOI: 10.1002/adma.202102988

(15) Loewe, R. S.; Ewbank, P. C.; Liu, J.; Zhai, L.; McCullough, R. D., Regioregular, Head-to-Tail Coupled Poly(3-alkylthiophenes) Made Easy by the GRIM Method: Investigation of the Reaction and the Origin of Regioselectivity. *Macromolecules* **2001**, *34* (13), 4324-4333. DOI: 10.1021/ma001677+

(16) Stefan, M. C.; Javier, A. E.; Osaka, I.; McCullough, R. D., Grignard Metathesis Method (GRIM): Toward a Universal Method for the Synthesis of Conjugated Polymers. *Macromolecules* **2008**, *42* (1), 30-32. DOI: 10.1021/ma8020823

(17) Liang, Z.; Neshchadin, A.; Zhang, Z.; Zhao, F.-G.; Liu, X.; Yu, L., Stille polycondensation: a multifaceted approach towards the synthesis of polymers with semiconducting properties. *Polym. Chem.* **2023**, *14* (40), 4611-4625. DOI: 10.1039/d3py00815k

(18) Bernoulli trial. https://en.wikipedia.org/wiki/Bernoulli_trial

(19) Wilson, E. B., Probable Interference, the Law of Succession, and Statistical Interference. *JASA* **1927**, *22* (158), 209-212. DOI: 10.1080/01621459.1927.10502953

(20) Generalized linear model. https://en.wikipedia.org/wiki/Generalized_linear_model

(21) Moro, S.; Siemons, N.; Drury, O.; Warr, D. A.; Moriarty, T. A.; Perdigao, L. M. A.; Pearce, D.; Moser, M.; Hallani, R. K.; Parker, J.; McCulloch, I.; Frost, J. M.; Nelson, J.; Costantini, G., The Effect of Glycol Side Chains on the Assembly and Microstructure of Conjugated Polymers. *ACS Nano* **2022**, *16* (12), 21303-21314. DOI: 10.1021/acsnano.2c09464

(22) Moro, S.; Spencer, S. E. F.; Lester, D. W.; Nubling, F.; ommer, M.; Costantini, G., Molecular-Scale Imaging Enables Direct Visualization of Molecular Defects and Chain Structure of Conjugated Polymers. *ACS Nano* **2024**, *18* (18), 11655-11664. DOI: 10.1021/acsnano.3c10842

(23) Griggs, S.; Marks, A.; Meli, D.; Rebetez, G.; Bardagot, O.; Paulsen, B. D.; Chen, H.; Weaver, K.; Nugraha, M. I.; Schafer, E. A.; Tropp, J.; Aitchison, C. M.; Anthopoulos, T. D.; Banerji, N.; Rivnay, J.; McCulloch, I., The effect of residual palladium on the

performance of organic electrochemical transistors. *Nat. Commun.* **2022**, *13* (1), 7964. DOI: 10.1038/s41467-022-35573-y

(24) Quill, T. J.; LeCroy, G.; Marks, A.; Hesse, S. A.; Thiburce, Q.; McCulloch, I.; Tassone, C. J.; Takacs, C. J.; Giovannitti, A.; Salleo, A., Charge Carrier Induced Structural Ordering And Disordering in Organic Mixed Ionic Electronic Conductors. *Adv. Mater.* **2024**, *36* (15), 2310157. DOI: 10.1002/adma.202310157

(25) Rivnay, J.; Leleux, P.; Ferro, M.; Sessolo, M.; Williamson, A.; Koutsouras, D. A.; Khodagholy, D.; Ramuz, M.; Strakosas, X.; Owens, R. M.; Benar, C.; Badier, J.-M.; Bernard, C.; Malliaras, G. G., High-performance transistors for bioelectronics through tuning of channel thickness. *Sci. Adv.* **2015**, *1* (4), e1400251. DOI: 10.1126/sciadv.1400251

(26) Bernards, D. A.; Malliaras, G. G., Steady-State and Transient Behavior of Organic Electrochemical Transistors. *Adv. Funct. Mater.* **2007**, *17* (17), 3538-3544. DOI: 10.1002/adfm.200601239

(27) Xu, Y.; Li, Y.; Li, S.; Balestra, F.; Ghibaudo, G.; Li, W.; Lin, Y.-F.; Sun, H. ; Wan, J.; Wang, X.; Guo, Y.; Shi, Y.; Noh, Y.-Y., Precise Extraction of Charge Carrier Mobility for Organic Transistors. *Adv. Funct. Mater.* **2020**, *30* (20), 1904508. DOI: 10.1002/adfm.201904508

(28) Friedlein, J. T.; McLeod, R. R.; Rivnay, J., Device physics of organic electrochemical transistors. *Org. Electron.* **2018**, *63*, 398-414. DOI: 10.1016/j.orgel.2018.09.010

(29) Shahi, M.; Le, V. N.; Espejo, P. A.; Alsufyani, M.; Kousseff, C. J.; McCulloch, I.; Paterson, A. F., The organic electrochemical transistor conundrum when reporting a mixed ionic-electronic transport figure of merit. *Nat. Mater.* **2024**, *23*, 2-8. DOI: 10.1038/s41563-023-01672-4

(30) Giovannitti, A.; Sbircea, D. T.; Inal, S.; Nielsen, C. B.; Bandiello, E.; Hanifi, D. A.; Sessolo, M.; Malliaras, G. G.; McCulloch, I.; Rivnay, J., Controlling the mode of operation of organic transistors through side-chain engineering. *Proc. Natl. Acad. Sci. U.S.A.* **2016**, *113* (43), 12017-12022. DOI: 10.1073/pnas.1608780113

(31) Moser, M.; Savagian, L. R.; Savva, A.; Matta, M.; Ponder, J. F.; Hidalgo, T. C.; Ohayon, D.; Hallani, R.; Reisjalali, M.; Troisi, A.; Wadsworth, A.; Reynolds, J. R.; Inal, S.; McCulloch, I., Ethylene Glycol-Based Side Chain Length Engineering in Polythiophenes and its Impact on Organic Electrochemical Transistor Performance. *Chem. Mater.* **2020**, *32* (15), 6618-6628. DOI: 10.1021/acs.chemmater.0c02041

(32) Schmode, P.; Savva, A.; Kahl, R.; Ohayon, D.; Meichsner, F.; Dolynchuk, O.; Thurn-Albrecht, T.; Inal, S.; Thelakkat, M., The Key Role of Side Chain Linkage in Structure Formation and Mixed Conduction of Ethylene Glycol Substituted Polythiophenes. *ACS Appl. Mater. Interfaces* **2020**, *12* (11), 13029-13039. DOI: 10.1021/acsami.9b21604

(33) Moser, M.; Hidalgo, T. C.; Surgailis, J.; Gladisch, J.; Ghosh, S.; Sheelamanthula, R.; Thiburce, Q.; Giovannitti, A.; Salleo, A.; Gasparini, N.; Wadsworth, A.; Zozoulenko, I.; Berggren, M.; Stavrinidou, E.; Inal, S.; McCulloch, I., Side Chain Redistribution as a Strategy to Boost Organic Electrochemical Transistor Performance and Stability. *Adv. Mater.* **2020**, *32* (37), e2002748. DOI: 10.1002/adma.202002748

(34) Moser, M.; Wang, Y.; Hidalgo, T. C.; Liao, H.; Yu, Y.; Chen, J.; Duan, J.; Moruzzi, F.; Griggs, S.; Marks, A.; Gasparini, N.; Wadsworth, A.; Inal, S.; McCulloch, I.; Yue, W., Propylene and butylene glycol: new alternatives to ethylene glycol in conjugated polymers for bioelectronic applications. *Mater. Horiz.* **2022**, *9*, 973-980. DOI: 10.1039/d1mh01889b

(35) Chen, S. E.; Flagg, L. Q.; Onorato, J. W.; Richter, L. J.; Guo, J.; Luscombe, C. K.; Ginger, D. S., Impact of varying side chain structure on organic electrochemical transistor performance: a series of oligoethylene glycol-substituted polythiophenes. *J. Mater. Chem. A* **2022**, *10* (19), 10738-10749. DOI: 10.1039/d2ta00683a

(36) Tseng, H.-S.; Puangniyom, T.; Chang, C.-Y.; Janardhanan, J. A.; Yu, H.-h.; Chen, W.-C.; Chueh, C.-C.; Hsiao, Y.-S., Strategically tailoring ethylene glycol side chains with bridged-carbonyl ester in polythiophene-based organic electrochemical transistors for bioelectronics. *J. Chem. Eng.* **2024**, *486*, 150371. DOI: 10.1016/j.cej.2024.150371

(37) Peng, Z.; Ye, L.; Ade, H., Understanding, quantifying, and controlling the molecular ordering of semiconducting polymers: from novices to experts and amorphous to perfect crystals. *Mater. Horiz.* **2022**, *9* (2), 577-606. DOI: 10.1039/d0mh00837k

(38) French, A. C.; Thompson, A. L.; Davis, B. G., High-purity discrete PEG-oligomer crystals allow structural insight. *Angew. Chem. Int. Ed. Engl.* **2009**, *48* (7), 1248-1252. DOI: 10.1002/anie.200804623
